# Long-read genome sequencing of bread wheat facilitates disease resistance gene cloning

**DOI:** 10.1038/s41588-022-01022-1

**Published:** 2022-03-14

**Authors:** Naveenkumar Athiyannan, Michael Abrouk, Willem H. P. Boshoff, Stéphane Cauet, Nathalie Rodde, David Kudrna, Nahed Mohammed, Jan Bettgenhaeuser, Kirsty S. Botha, Shannon S. Derman, Rod A. Wing, Renée Prins, Simon G. Krattinger

**Affiliations:** 1 Center for Desert Agriculture, Biological and Environmental Science and Engineering Division (BESE), King Abdullah University of Science and Technology (KAUST), Thuwal, Saudi Arabia; 2 Department of Plant Sciences, University of the Free State, Bloemfontein, South Africa; 3 INRAE-CNRGV French Plant Genomic Resource Center, Castanet-Tolosan, France; 4 Arizona Genomics Institute, School of Plant Sciences, University of Arizona, Tucson, AZ USA; 5 CenGen (Pty) Ltd, Worcester, South Africa; 6 Department of Genetics, Stellenbosch University, Stellenbosch, South Africa

**Keywords:** Plant genetics, Genomics

## Abstract

The cloning of agronomically important genes from large, complex crop genomes remains challenging. Here we generate a 14.7 gigabase chromosome-scale assembly of the South African bread wheat (*Triticum aestivum*) cultivar Kariega by combining high-fidelity long reads, optical mapping and chromosome conformation capture. The resulting assembly is an order of magnitude more contiguous than previous wheat assemblies. Kariega shows durable resistance to the devastating fungal stripe rust disease^1^. We identified the race-specific disease resistance gene *Yr27*, which encodes an intracellular immune receptor, to be a major contributor to this resistance. *Yr27* is allelic to the leaf rust resistance gene *Lr13*; the Yr27 and Lr13 proteins show 97% sequence identity^2,3^. Our results demonstrate the feasibility of generating chromosome-scale wheat assemblies to clone genes, and exemplify that highly similar alleles of a single-copy gene can confer resistance to different pathogens, which might provide a basis for engineering *Yr27* alleles with multiple recognition specificities in the future.

## Abstract (web summary)

Chromosome-scale genome assembly of the South African bread wheat (*Triticum aestivum*) cultivar Kariega facilitates the cloning of the stripe rust resistance gene *Yr27*.

## Main

**Fig. 1:**
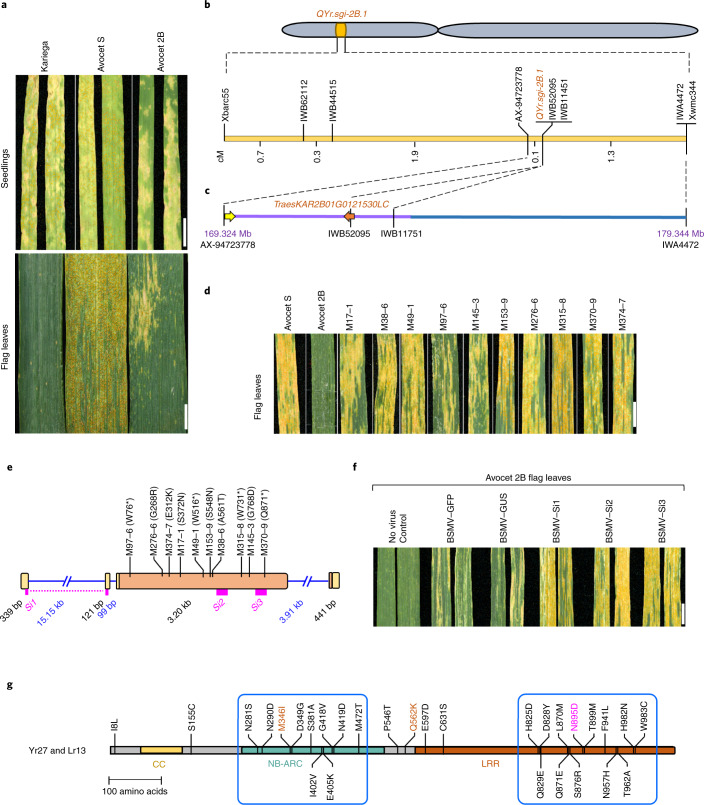
Assembly-guided cloning of *QYr.sgi-2B.1*. **a**, Stripe rust symptoms on Kariega, Avocet S and Avocet 2B in seedlings (upper panel) and adult plants (lower panel). Scale bars, 1 cm. **b**, Genetic map of the *QYr.sgi-2B.1* region. Numbers between markers indicate genetic distances. **c**, Physical interval of the *QYr.sgi-2B.1* region. Numbers indicate positions (in megabases) in the Kariega 2B pseudomolecule. The interval contained 93 candidate genes that were determined based on the annotated high-confidence gene models (92 high-confidence genes) and the NLR annotation. The NLR annotation identified one additional gene (*TraesKAR2B01G0121530LC*) that was annotated as a low-confidence gene. The two arrows indicate NLR-encoding genes that were prioritized for gene validation. Purple and blue bars indicate two primary HiFi contigs (47.7 Mb and 19.5 Mb, respectively) that spanned the target interval. **d**, Stripe rust inoculation on Avocet 2B and ten independent EMS mutants that lost the *QYr.sgi-2B.1-*mediated resistance. Scale bar, 1 cm. **e**, Gene structure of *QYr.sgi-2B.1*. Boxes, exons; lines, introns; yellow boxes, 5′ UTR and 3′ UTR; orange boxes, coding sequence. Positions of the corresponding amino acid changes in the ten loss-of-function mutants are indicated. *Si1*, *Si2* and *Si3* denote three VIGS constructs. **f**, Representative images showing the results of the VIGS experiment. The control ‘no virus’ contained no BSMV. BSMV–GFP and BSMV–GUS denote controls with a *GFP* or *GUS* silencing construct, respectively. BSMV–Si1, BSMV–Si2 and BSMV–Si3 denote three different silencing constructs that target *QYr.sgi-2B.1*. The specificity of *Si1–Si3* was evaluated using the Kariega assembly. The image shows flag leaves 15 days after inoculation with an avirulent *Pst* isolate. Chlorotic areas on the GUS and GFP controls represent virus symptoms. Scale bar, 1 cm. **g**, Comparison of the Yr27 and Lr13 protein sequences. The first residue is the one present in Yr27, and the second residue is the one present in Lr13. Blue boxes indicate regions with increased polymorphism density. The two amino acids shown in brown have been found to determine the *Lr13*-specificity^2^. The residue shown in magenta is found only in Yr27.

Circular consensus sequencing (CCS)^4^ represents a recent technological breakthrough in DNA sequencing that circumvents the hitherto negative relationship between read length and accuracy, enabling the production of genome assemblies with greatly improved completeness and contiguity^5,6^. However, the large (approximately 16 gigabase (Gb)), repeat-rich genome of polyploid bread wheat remains a challenge for genome sequencing and gene cloning projects. A comparison of the genomes of various bread wheat cultivars demonstrated their high tolerance for extensive structural rearrangements, introgressions from wild wheat relatives and differences in gene content^7,8^, highlighting the need to generate genomic resources from specific donor wheat lines to guide the cloning of agronomically important genes. Stripe rust (or yellow rust), caused by the fungal pathogen *Puccinia striiformis* f. sp. *tritici* (*Pst*), is of increasing concern for global wheat production^9^. Of the 83 yellow rust resistance (*Yr*) genes described in the wheat gene pool, only nine have been cloned so far^10^, precluding knowledge-guided global deployment of *Yr* genes based on sequences and molecular mechanisms. Kariega, a South African spring bread wheat cultivar released in 1993, shows high levels of adult plant stripe rust resistance (Fig. 1a). Despite its extensive use in wheat production and as a parent in breeding programs, Kariega remains resistant to all *Pst* races prevalent in South Africa. The resistance of Kariega to stripe rust is conferred by three quantitative trait loci (QTLs): *QYr.sgi-2B.1* on chromosome arm 2BS; *QYr.sgi-4A.1* on chromosome arm 4AL; and the durable stripe rust resistance gene *Yr18* on chromosome arm 7DS, which encodes an ATP-binding cassette (ABC) transporter^1,11^.

**Extended Data Fig. 1:**
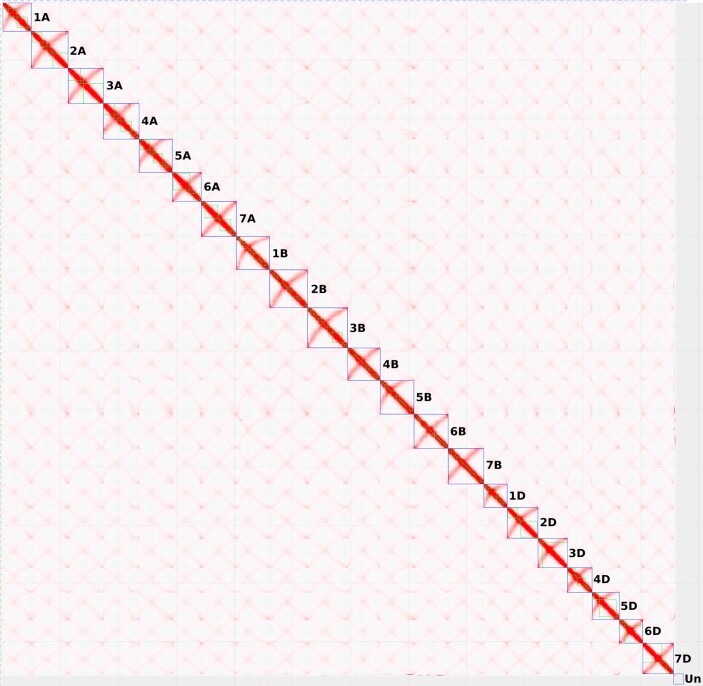
Chromosome contact maps across the 21 pseudomolecules of the Kariega assembly. Green squares indicate individual hybrid scaffolds and blue boxes pseudomolecules.

**Extended Data Fig. 2:**
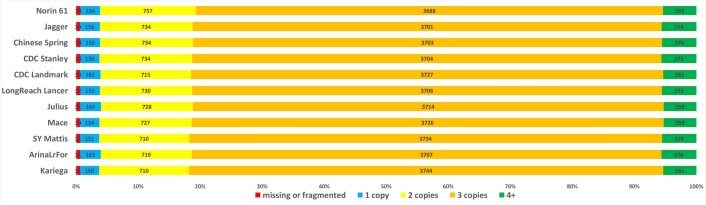
Number of universal single-copy orthologs that were missing or fragmented, present in a single copy, or present in multiple copies. The result of the Kariega assembly is compared to the chromosome-scale assemblies of the 10+ Wheat Genomes Project.

**Extended Data Fig. 3:**
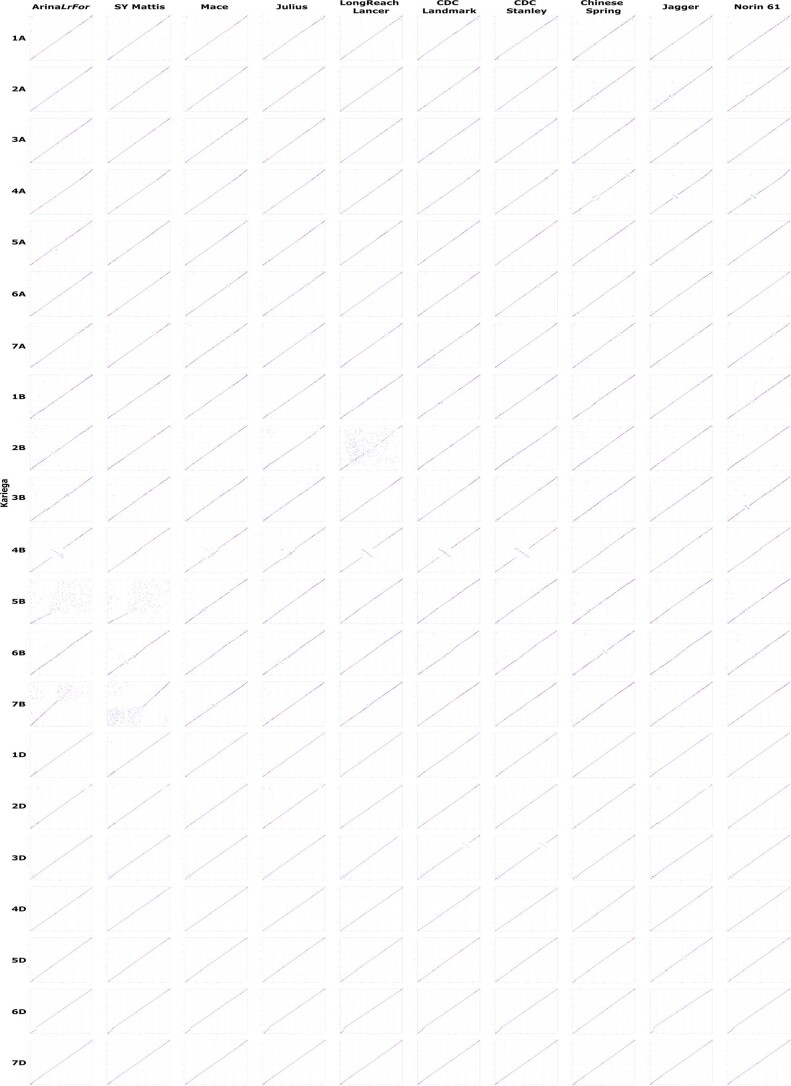
Comparison of the Kariega pseudomolecule assembly with the Chinese Spring IWGSC RefSeq v2.1 and the chromosome-scale assemblies of the 10+ Wheat Genomes Project.

To facilitate the cloning of the remaining stripe rust resistance genes from Kariega, we generated a de novo genome assembly. First, we generated PacBio CCS (HiFi) reads corresponding to approximately 34-fold coverage (Supplementary Table 1) that we assembled using hifiasm^12^, resulting in an assembly length of 14.66 Gb with a contig N50 length of 30.22 megabases (Mb) (Table 1 and Supplementary Table 2). For comparison, contig N50s from previous whole-genome bread wheat assemblies based on short-read sequencing or PacBio continuous long-read (CLR) sequencing ranged from 49 to 233 kilobases (kb)^7,13,14^; thus, the Kariega assembly represents a ~130–600-fold improvement. The assembly took 43 h of computing time on 48 central processing unit (CPU) cores and ~550 gigabytes of random access memory (RAM). Next we generated hybrid scaffolds using a direct label and stain (DLS) optical map (Supplementary Table 3), resulting in the assembly of 324 hybrid scaffolds with a combined length of 14.5 Gb and an N50 of 204.3 Mb. The longest hybrid scaffold was 627.2 Mb and covered 99% of chromosome 3D (Supplementary Table 3). The final 14.68 Gb chromosome-scale assembly was produced by integrating chromosome conformation capture data (Omni-C; Extended Data Fig. 1). About 98.5% (14.45 Gb) of the assembly comprised 21 pseudomolecules, with the remaining 0.22 Gb combined into an unanchored pseudochromosome (Table 1). We validated the concordance of the assembly by mapping the optical map onto the Kariega pseudomolecules and found no major discrepancies. We recovered 99.4% of the 4,896 Poales single-copy core genes (BUSCO v.5.0.0)^15^ in the Kariega assembly, with 96.3% being duplicated (Extended Data Fig. 2). This underscores the high contiguity, completeness and accuracy of the assembly. Comparison of the Kariega assembly with previous chromosome-scale bread wheat assemblies revealed high collinearity (Extended Data Fig. 3). We annotated the Kariega assembly with support from transcriptome (Supplementary Table 4) and isoform (Supplementary Table 5) sequencing from six different tissues, and defined 116,838 high-confidence gene models.

**Table 1:** Statistics of the Kariega genome assembly and annotation

Genomic feature	Value
*Length of HiFi assembly*	14.66 Gb
Number of contigs	5,055
Length of contig N50	30.22 Mb
Length of contig N90	5.5 Mb
*Length of hybrid assembly*^a^	14.68 Gb
*Length of hybrid scaffolds*	14.46 Gb
Number of hybrid scaffolds	324
Length of hybrid scaffold N50	204.26 Mb
Gap size	18.8 Mb (0.13%)
*Length of pseudomolecule assembly*	14.68 Gb
*Total length of anchored pseudomolecules*	14.45 Gb
Number of anchored hybrid scaffolds or contigs	717
Gap size	11.6 Mb (0.08%)
Number of high-confidence genes	110,383
*Total length of unanchored chromosome*	224.01 Mb
Number of unanchored hybrid scaffolds or contigs	3,910
Gap size	8.1 Mb (3.6%)
Number of high-confidence genes	6,455
*BUSCO*	
Complete	99.4%
Duplicated	96.3%
Fragmented	0.1%
Missing	0.5%

^a^Includes all hybrid scaffolds and remaining contigs that were not integrated into hybrid scaffolds.

To explore the phenotype conferred by *QYr.sgi-2B.1* in the absence of the other two stripe rust resistance QTLs, we backcrossed Kariega with the susceptible spring wheat cultivar Avocet S. The resulting backcross line, Avocet S + *QYr.sgi-2B.1* (herein referred to as Avocet 2B), showed moderate seedling resistance and strong adult plant resistance to the South African *Pst* isolate 6E22A+ (Fig. 1a), but susceptibility to race 30E142A+, which was recently reported in Zimbabwe^16^, indicating that *QYr.sgi-2B.1* is a race-specific disease resistance gene. Using an Avocet 2B × Avocet S F_2_ population, we mapped *QYr.sgi-2B.1* to a genetic interval of 1.4 centimorgans (cM) (Fig. 1b), corresponding to a physical region of 10.02 Mb in the Kariega assembly and containing 93 candidate genes (Fig. 1c). Among these were two genes encoding nucleotide-binding leucine-rich repeat (NLR) proteins, which are intracellular immune receptors associated with plant immunity^17^. To confirm the identity of *QYr.sgi-2B.1*, we screened an Avocet 2B mutant population induced with ethyl methanesulfonate (EMS), yielding ten independent mutants that had all lost the *QYr.sgi-2B.1-*mediated stripe rust resistance (Fig. 1d). All ten mutants harbored an EMS-type (G/C to A/T), nonsynonymous mutation in one of the two NLR genes (*TraesKAR2B01G0121530LC*) (Fig. 1e). Silencing of this NLR gene in Avocet 2B by virus-induced gene silencing (VIGS) greatly reduced stripe rust resistance (Fig. 1f). In summary, the genetic mapping, ten independent mutants and gene silencing confirm *TraesKAR2B01G0121530LC* to be the causal gene.

**Extended Data Fig. 4:**
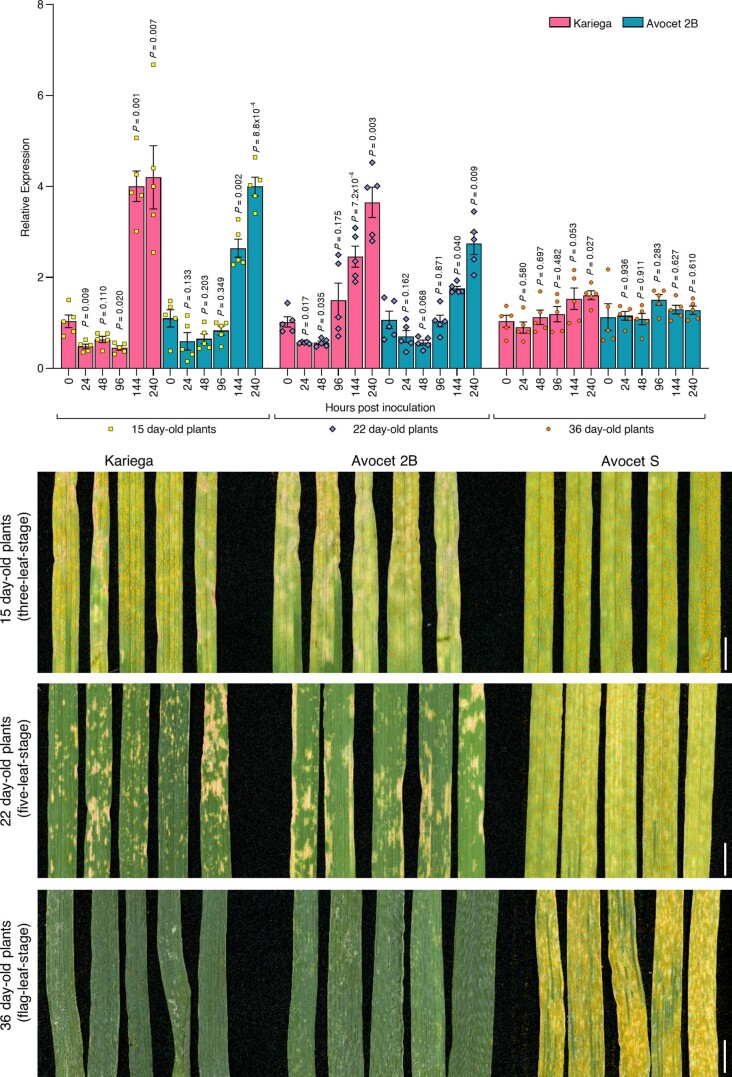
*QYr.sgi-2B.1* expression in Kariega and Avocet 2B after inoculation with an avirulent *Pst* isolate (6E22A+). Values and error bars represent means and standard errors of five biological replicates, respectively. Statistical analysis was done using a two-tailed t-test (compared to 0 hours post inoculation). The lower panel shows representative images of leaves harvested at the time of sampling (15 days after inoculation). Scale bar = 1 cm.

**Extended Data Fig. 5:**
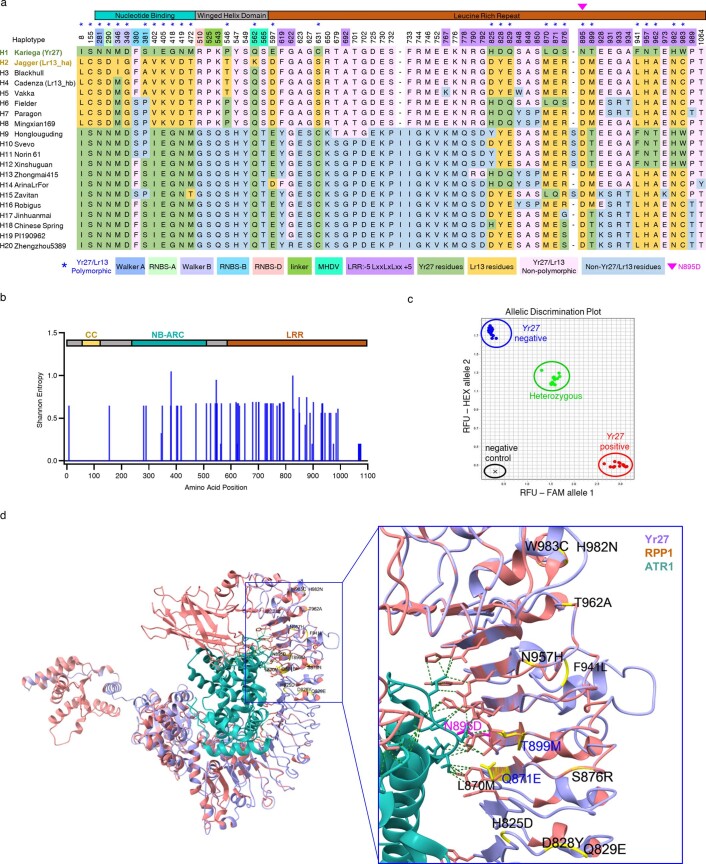
Yr27 haplotype analysis, diversity, and homology modelling. **a**, Polymorphic residues encoded by *Yr27* alleles present in various wheat cultivars. Blue asterisks indicate residues that are polymorphic between Lr13 and Yr27. Functional motifs are indicated in different colors. **b**, Shannon entropy plot showing amino acid diversity across Yr27 based on the alignment of 20 different haplotypes. **c**, *Yr27*-specific KASP marker tested on *Yr27* positive, *Yr27* negative and heterozygous wheat plants (Avocet 2B x Avocet S F2 population). **d**, Yr27 model superimposed to the *Arabidopsis thaliana* RPP1-ATR1 cryo-EM structure. The close-up view shows the RPP1-ATR1 contact interface in the LRR domain. The labeled residues are the ones that were polymorphic between Lr13 and Yr27. The three residues Q871, N895 and T899 are predicted to locate on the inner surface of the LRR domain and the corresponding residues in RPP1 have been shown to form polar interactions with the ATR1 effector (indicated by green dashed lines).

Next we confirmed the structure of the *QYr.sgi-2B.1* gene using Kariega Iso-Seq reads. The *QYr.sgi-2B.1* gene spans 23.2 kb and consists of four exons, the first two of which correspond to the 5′ untranslated region (UTR). Notably, the first intron is 15.15 kb long (Fig. 1e). The predicted coding sequence with a length of 3,219 base pairs (bp) encodes a protein of 1,072 amino acids with an N-terminal coiled-coil domain, a central NB-ARC domain and a carboxy-terminal leucine-rich repeat (LRR) domain. The *QYr.sgi-2B.1* coding sequence was identical in Avocet 2B and Kariega, but polymorphic in Avocet S, confirming that the gene in Avocet 2B was introgressed from Kariega. *QYr.sgi-2B.1* transcript levels increased approximately fourfold in seedlings after inoculation with an avirulent *Pst* isolate, but were not induced or only moderately induced in adult plants (Extended Data Fig. 4). Several *Yr* genes were genetically assigned to chromosome arm 2BS, including *Yr27* and *Yr31* (ref. ^18^). The *QYr.sgi-2B.1* coding sequence was identical to that of an NLR gene amplified from several *Yr27* reference stocks, including the wheat lines Selkirk, Kauz, Kubsa, Opata 85 and Avocet *Yr27* (ref. ^19^), indicating that *QYr.sgi-2B.1* is *Yr27*. Wheat cultivars containing *Yr27* have been widely deployed in regions of South Asia and East Africa prone to stripe rust^9,19^, resulting in the emergence of *Pst* races that are virulent to *Yr27*. The phenotypic expression of *Yr27* (previously known as *YrSk*) at the seedling stage is influenced by the environment and genetic background^19,20^. We discovered that *Yr27* is allelic to the recently cloned leaf rust resistance gene *Lr13* (refs. ^2,3^). NLRs often form complex gene clusters, and it has been found that different members of such allelic series can confer resistance to various fungal pathogens. For example, the powdery mildew resistance gene *TmMla1* from diploid einkorn wheat (*Triticum monococcum*)^21^ and the *Sr33* stem rust resistance gene from the wild wheat progenitor *Aegilops tauschii*^22^ are both orthologous to *Mla*, a complex cluster of NLR genes originally described in barley (*Hordeum vulgare*)^23^, and encode proteins with 86% sequence identity. By contrast, *Yr27* and *Lr13* represent alleles of a single-copy gene (Fig. 1c), and the protein versions that they encode are 97.3% identical, differing in only 29 of 1,072 amino acids (Fig. 1g). Most of the polymorphisms are clustered in two regions: a 192 amino acid stretch in the NB-ARC domain and a 159 amino acid region in the LRR domain (Fig. 1g). Analysis of Shannon entropy, an unbiased measure used to predict rapidly evolving residues^24^, revealed a higher level of amino acid diversity in the LRR domain among Yr27/Lr13 protein variants present in various wheat cultivars compared with the diversity in the N-terminal coiled-coil and NB-ARC domains (Extended Data Fig. 5a,b). *Lr13* results not only in leaf rust resistance, but also in hybrid necrosis in certain genetic backgrounds^2,3^. Two amino acids in the NB-ARC domain (Ile 346 and Lys 562) of Lr13 are critical for leaf rust resistance specificity^2^. At the equivalent positions, Yr27 had the same residues as the Lr13_hb haplotype, which lacked resistance to leaf rust but retained the hybrid necrosis phenotype (Extended Data Fig. 5a). A comparison with publicly available wheat sequences^7,13,25,26^ revealed one amino acid (Asn 895) that was found only in Yr27 (Fig. 1g), which we turned into a *Yr27*-specific molecular marker for breeding (Extended Data Fig. 5c). Protein modeling predicted that three of the polymorphic residues in the highly variable 159 amino acid region of the Yr27 LRR domain (including the unique Asn 895 amino acid) coincide with the inner surface of the LRR domain (Extended Data Fig. 5d). The corresponding residues of the *Arabidopsis thaliana* NLR immune receptor RPP1 contribute to the interaction of RPP1 with the ATR1 pathogen effector^27^. Together, these data might indicate that Yr27 is a highly variable NLR and that the variable stretch in the LRR domain might be important to define recognition specificity.

**Fig. 2:**
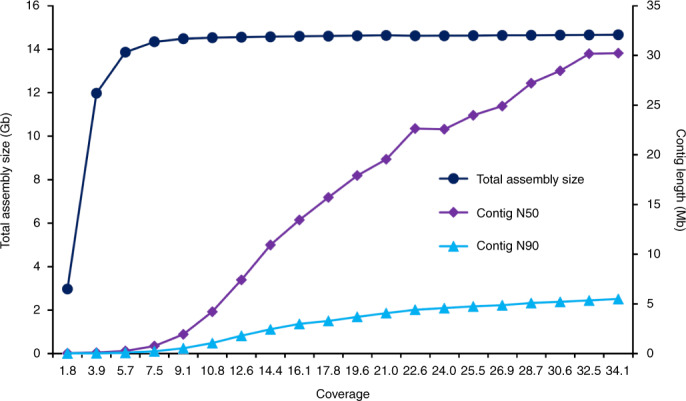
Total assembly size, contig N50 length and contig N90 length of 20 individual Kariega assemblies. Assemblies were produced using subsets of the PacBio HiFi data.

In summary, we have demonstrated the feasibility of generating chromosome-scale wheat assemblies from any wheat line to guide gene cloning projects. The Kariega assembly was completed in a short period of time (<4 months) and for a fraction of the cost of previous chromosome-scale wheat assemblies. Notably, we produced assemblies with lengths greater than 14 Gb from subsets of PacBio HiFi reads, corresponding to ~7.5-fold and ~10.8-fold coverage, with contig N50 lengths of 0.76 Mb and 4.2 Mb, respectively, which might be sufficient for most gene cloning projects (Fig. 2 and Supplementary Table 2). Our approach eliminates the need for the time-consuming, laborious complexity reduction required with previous genomics-based gene cloning methods in wheat^28–31^. NLR genes, including *Yr27*, are often associated with rapid resistance breakdown when widely deployed. Our results show that the race-specific *Yr27* gene is a component of the quantitative and durable stripe rust resistance seen in Kariega. Similarly, the race-specific *Lr14a* leaf rust resistance gene, which encodes a membrane-bound ankyrin repeat protein, was recently identified as one of several QTLs that contribute to the broad-spectrum and durable leaf rust resistance in the Swiss winter wheat cultivar Forno^32^. The longevity of the rust resistances in Kariega and Forno might be the result of optimal ‘stacks’ of partial broad-spectrum resistance genes (such as *Lr34* (also known as *Yr18*)) and race-specific *R* genes, indicating that the optimal deployment of race-specific resistance genes, in combination with partial broad-spectrum resistance genes, can result in near-complete and long-lasting disease resistance. Most notably, *Yr27* is an allele of *Lr13*, indicating that near-identical alleles can confer race-specific resistance to different pathogens. This result may be exploited to engineer NLR versions with multipathogen recognition specificity in the future.

## Methods

### Plant materials

**Extended Data Fig. 6:**
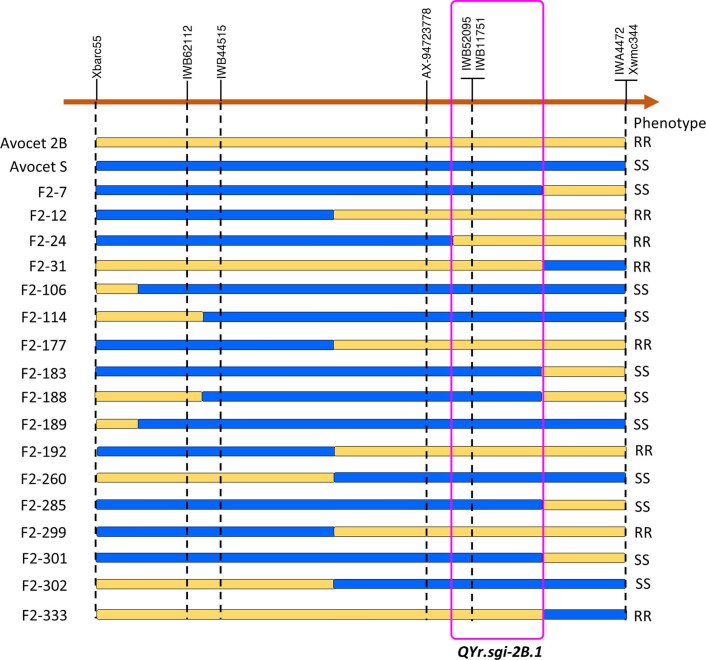
Graphical representation of critical recombinants of the Avocet 2B x Avocet S F2 population.

The bread wheat cultivars Kariega and Avocet S, and the near-isogenic line Avocet S + *QYr.sgi-2B.1* (Avocet 2B) were used to clone *Yr27*. Avocet 2B was developed by selfing a BC4F2 plant that was selected from backcross material derived from crossing *QYr.sgi-2B.1* from Kariega into the susceptible background of Avocet S^1^. The resultant BC4F6 material was designated as Avocet 2B. The molecular markers *Xbarc55* and *Xwmc344* were used to verify the presence of *QYr.sgi-2B.1* in Avocet 2B. In addition, Kariega, Avocet S and Avocet 2B were genotyped with a wheat 90K iSelect single nucleotide polymorphism (SNP) array^33^ and a Wheat Breeders’ 35K Axiom Array^34^, which confirmed that only the *QYr.sgi-2B.1* region, and none of the other Kariega stripe rust QTLs, has been introgressed into Avocet 2B (Supplementary Table 6). An F_2_ mapping population (Avocet 2B × Avocet S; *n* = 345) segregating for a single dominant stripe rust resistance gene (R:S = 254:91, χ^2^ = 0.348, *P* = 0.554) was genotyped with the *QYr.sgi-2B.1* flanking markers^1^ *Xbarc55* and *Xwmc344* to identify recombinants. Identified recombinants were genotyped with additional Kompetitive Allele Specific PCR (KASP) markers derived from polymorphic iSelect and Axiom 35K SNPs between Avocet S and Avocet 2B (Extended Data Fig. 6 and Supplementary Table 7)^35^. The following *Yr27*-carrying lines were obtained from the University of the Free State (UFS) and the Agricultural Research Council - Small Grain in South Africa (SA): Selkirk (UFS31b), Kubsa (SA9399), Kauz (SA6643), Opata 85 (SA8997) and Avocet *Yr27* (*Yr27*/6*AvS; UFS31a).

### HiFi library preparation and sequencing

High molecular weight DNA was extracted from dark-treated, young Kariega leaves following the protocols of refs. ^36,37^. DNA purity was assessed on a NanoDrop NP-1000 spectrophotometer (NanoDrop Technologies), DNA concentration was measured with a Qubit dsDNA high-sensitivity assay and DNA size was validated by pulsed-field gel electrophoresis (PFGE). Ten micrograms of DNA was sheared to the appropriate size range (10–30 kb) using a Covaris g-TUBE for the construction of PacBio HiFi sequencing libraries, followed by bead purification with PB Beads (PacBio). Sequencing libraries were constructed following the manufacturer’s protocol using a SMRTbell Express Template Prep Kit 2.0. Libraries were quantified using the Qubit dsDNA high-sensitivity assay, and size was checked on a Femto Pulse System (Agilent). Sequencing was performed on PacBio Sequel II systems in CCS mode for 30 h.

### Optical map production

Grains of Kariega were germinated and grown in the dark on wet filter paper for 4 days at 4 °C and 3 days at 25 °C. One gram of fresh root meristem tissue was fixed and treated according to the Plant DNA Isolation Kit protocol (Bionano Genomics) to recover high molecular weight DNA embedded in agarose plugs. Plugs containing DNA were washed in wash buffer (Bionano Genomics) and Tris-EDTA (TE). After a purification step using a PFGE system (Bio-Rad) to remove smaller DNA fragments (90 min; 5 V cm^–1^; angle, 120 °C; initial switch time, 1 s; final switch time, 3 s; linear ramping), we proceeded to DNA release using gelase followed by a dialysis according to the protocol (Bionano Genomics). DNA quantity and size were estimated using Qubit (Invitrogen) and PFGE (24 h; 6 V cm^–1^; angle, 120 °C; initial switch time, 60 s; final switch time, 120 s; linear ramping). DNA was labeled using the DLS protocol (Bionano Genomics). Labeled molecules were produced using the Saphyr System (Saphyr Chip G1.2). Data processing was performed using the Bionano Solve v.3.6 software (https://bionanogenomics.com/support/software-downloads).

### Omni-C library preparation and sequencing

The Omni-C library was prepared using the Dovetail Omni-C Kit according to the manufacturer’s protocol. In brief, chromatin was fixed in place in the nucleus. Fixed chromatin was digested with DNase I and then extracted. Chromatin ends were repaired and ligated to a biotinylated bridge adapter, before proximity ligation of adapter containing ends. After proximity ligation, crosslinks were reversed and the DNA was purified from proteins. Purified DNA was treated to remove biotin that was not internal to ligated fragments. Four sequencing libraries were generated using Illumina-compatible adapters. Biotin-containing fragments were isolated using streptavidin beads before PCR enrichment of the library. The four libraries were sequenced on an Illumina HiSeq X platform to generate 834 million 2 × 150 bp read pairs.

### RNA-Seq and Iso-Seq library preparation and sequencing

Total RNA was extracted from seedlings, seedlings at dusk, flag leaves, grains, roots and spikes. In brief, 100 mg of ground powder from each tissue was used for RNA isolation using a Maxwell RSC Plant RNA Kit (AS1500) with a Maxwell RSC48 instrument as indicated in the kit protocol (Promega). Around 20 Gb of 2 × 150 bp reads were generated for each sample. The Iso-Seq SMRTbell library was constructed according to the standard isoform sequencing protocol (Pacific Biosciences, 101-763-800) using the NEBNext Single Cell/Low Input cDNA Synthesis & Amplification Module (New England Biolabs, E6421S) and the ProNex Size-Selective Purification System (Promega, NG2001) for size selection. In brief, 300 ng of total RNA from each of the six developmental stages was used as input for complementary DNA synthesis. Each sample was first barcoded and then subjected to cDNA amplification using 12 cycles. Purified cDNAs were pooled in equal molarity and then subjected to library preparation using the SMRTbell Express Template Prep Kit 2.0 (Pacific Biosciences, 100-938-900) following the Iso-Seq protocol previously referenced. The library was prepared for sequencing by annealing primer v4 with the Sequel II Binding Kit 2.1 and the Internal Control Kit 1.0 (Pacific Biosciences, 101-843-000). One SMRT Cell 8M (Pacific Biosciences, 101-389-001) was sequenced on the PacBio Sequel II system using the Sequencing Kit 2.0 (Pacific Biosciences, 101-820-200). The IsoSeq pipeline (v.3; https://github.com/PacificBiosciences/IsoSeq) was used for CCS generation, demultiplexing and clustering of the six datasets.

### Genome assembly and validation

The PacBio HiFi reads were assembled using hifiasm^12^ (v.0.11) with default parameters. Hybrid scaffolding incorporating the PacBio contigs and the optical map was performed using the hybridScaffold pipeline (Bionano Solve 3.6) with default parameters. For the pseudomolecule construction, the Omni-C reads were incorporated using Juicer tools^38^ (v.1.6) and 3D-DNA^39^ (v.180114). In brief, the preprocessing of the Omni-C reads was performed with juicer.sh (parameter: -s none). The ‘merged_nodups.txt’ output file corresponding to the Hi-C contacts with duplicates removed was subsequently used with run-asm-pipeline.sh (parameter: -r 0) as input to produce the ‘.hic’ and ‘.assembly’ files. These files were uploaded into Juicebox^40^ (v.1.11.08) to visualize the Hi-C map and for manual curation. As a final step, the script run-asm-pipeline-post-review.sh (default parameters) was used to save the final Hi-C contact map and to output the final Kariega assembly (21 pseudomolecules and 1 unanchored pseudochromosome). To validate the genome assembly, we remapped the optical map onto the pseudomolecule using the hybridScaffold pipeline (Bionano Solve 3.6), and the final pseudomolecules were compared with the recent bread wheat assemblies of Chinese Spring (IWGSC RefSeq v.2.1)^41^ and the assemblies of the 10+ Wheat Genomes Project^7^ using MashMap^42^ (v.2.0; parameter: -s 300000–pi 98).

### Gene model prediction

Gene model prediction was performed following the method described by Mascher et al.^5^ with minor modifications, combining transcriptomics data, protein homology and ab initio prediction. First, the RNA-Seq data from the six developmental stages were mapped to the reference assembly using STAR^43^ (v.2.7.0f; parameter:–outFilterMismatchNoverReadLmax 0.02) and assembled into transcripts with StringTie^44^ (v.2.1.4; parameter:–rf -m 150 -f 0.3 -t). Iso-Seq data were mapped using minimap2^45^ (v.2.17-r941; parameter: -ax splice -uf –secondary=no -C5), and the redundant isoforms were further collapsed into transcript loci using cDNA_Cupcake (http://github.com/Magdoll/cDNA_Cupcake; parameter:–dun-merge-5-shorter). The RNA-Seq and Iso-Seq transcripts were merged using StringTie (parameters:–merge -m 150) into a pool of candidate transcripts, and TransDecoder (v.5.5.0; https://github.com/TransDecoder/TransDecoder) was used to find potential open reading frames and to predict protein sequences within the candidate transcript set. For the protein homology evidences, we used the translated proteins from the projected gene annotation of the 10+ Wheat Genomes Project^7^, the IWGSC RefSeq v.2.1^41^ and the Triticeae protein sequences downloaded from the UniProt database (2021_03). All of the proteins were mapped against the Kariega assembly using GenomeThreader^46^ (v.1.7.1; parameters: -startcodon -finalstopcodon -species rice -gcmincoverage 70 -prseedlength 7 -prhdist 4 -gff3out). Then, we produced ab initio gene predictions using AUGUSTUS^47^ (v.3.4.0), GeneMark^48^ (v.4.38) and Fgenesh (v.8.0.0; http://www.softberry.com). In brief, AUGUSTUS gene prediction was performed using a model specifically trained according to Hoff and Stanke^49^ and a hints file generated using the previously mentioned Iso-Seq and RNA-Seq predictions. GeneMark was used with the option -ET and the intron coordinates calculated with the perl script star_to_gff.pl provided in the GeneMark package. For the Fgenesh prediction, the Kariega pseudomolecules were repeat masked using a de novo repeat library constructed with the Extensive de novo TE Annotator (EDTA) pipeline^50^ and the TREP database^51^ (v.19). Fgenesh annotation was performed with the specific *Triticum aestivum* matrix for the gene prediction. We used EVidenceModeler^52^ (v.1.1.1) to join all of the gene evidences from transcriptomics, protein alignments and ab initio predictions with weights adjusted according to the input source (Fgenesh, 2; AUGUSTUS, 1; GeneMark, 1; protein homology, 5; transcriptomics, 12). Finally, we performed two rounds of isoform and UTR prediction using the Program to Assemble Spliced Alignments (PASA) pipeline^53^ with default parameters. Gene models were classified as high-confidence or low-confidence genes according to criteria used by the International Wheat Genome Sequencing Consortium^13^ and by Mascher et al.^5^. In brief, protein-encoding gene models were considered complete when start and stop codons were present. A comparison with PTREP^51^, UniPoa (Poaceae database of annotated proteins from the UniProt database (2021_03)) and UniViri (Viridiplantae database) was performed using DIAMOND^54^ (v.2.0.9; parameters: -e 1e-10–query-cover 80–subject-cover 80). Gene candidates were further classified using the following criteria: a high-confidence gene model is complete with a hit in the UniViri database and/or in UniPoa and not PTREP; a low-confidence gene model is incomplete and has a hit in the UniViri or UniPoa database but not in PTREP, or the protein sequence is complete with no hit in UniViri, UniPoa or PTREP. Putative functional annotations were assigned to high-confidence and low-confidence genes using a protein comparison with the UniProt database (2021_03). The detection of putative NLR loci on each of the pseudomolecules was performed using the NLR-Annotator pipeline^55^ with default parameters.

### Stripe rust inoculations

Stripe rust inoculations at seedling and adult plant stages were performed with urediniospores of the South African *Pst* pathotype 6E22A+ (virulent to *Yr2*, *Yr6*, *Yr7*, *Yr8*, *Yr17*, *Yr25* and *YrA*; avirulent to *Yr1*, *Yr3a*, *Yr4a*, *Yr4b*, *Yr5*, *Yr9*, *Yr10*, *Yr15* and *Yr27*)^56^ and pathotype 30E142A+^16^ (virulent to *Yr2*, *Yr3a*, *Yr4a*, *Yr6*, *Yr7*, *Yr8*, *Yr9*, *Yr19*, *Yr25*, *Yr27* and *YrA;* avirulent to *Yr1*, *Yr4b*, *Yr5*, *Yr10*, *Yr15*, *Yr24*, *Yr32*, *YrCle*, *YrMor*, *YrSd*, *YrSp*, *YrSu* and *YrHVII)*. In brief, freshly propagated urediniospores were suspended in FC-43 oil (3M Fluorinert FC-43) and then sprayed onto the leaves using a high-pressure air sprayer. After inoculation, the plants were placed in a plastic box (56 × 39 × 42 cm), sprayed with Milli-Q water to maintain a high level of humidity and kept in the dark for 24 h at 4 °C. Then, plants were moved to a growth chamber with a 16/8 h day/night regime with the temperature set to 21/18 °C. At 15 days after inoculation, stripe rust phenotypes were recorded by scanning the leaves at 600 dots per inch on an Epson Perfection V850 Pro scanner.

### EMS mutagenesis

EMS mutagenesis of Avocet 2B was performed with a concentration of 0.6% EMS (Sigma-Aldrich, M0880). About 2,000 grains were soaked in water at 4 °C for 16 h, dried for 8 h on filter paper and incubated for 16 h with shaking at room temperature (23 °C) in the EMS solution. Next, the grains were washed three times for 45 min each and rinsed for 30 min under running tap water. Grains were pregerminated on humid filter paper in the dark at 4 °C for 48 h. The pregerminated grains were planted in 24-well trays (six grains per well) filled with Stender potting soil in a greenhouse illuminated with Heliospectra LX602C light-emitting diode (LED) grow lights (20/4 h day/night regime with temperature set to 21/18 °C). Single spikes of M0 plants were harvested, and at least ten M1 progenies per M0 plant were phenotyped at the flag leaf stage with *Pst* pathotype 6E22A+ to identify susceptible candidate mutant lines, which were validated in the M2 and M3 generations.

### Genetic map

Linkage analysis was performed using MapDisto 2.0^57^ with default parameters such as LOD (logarithm of the odds) threshold of 3.0, maximum recombination frequency of 0.3 and removal of loci with 10% missing data. Genetic distances were calculated using the Kosambi mapping function, and the map was created using MapChart^58^. A graphical representation of critical recombinants of the Avocet 2B × Avocet S population is shown in Extended Data Fig. 6.

### PCR conditions

A 20 μl PCR containing 100 ng of genomic DNA, 1× GoTaq Green Master Mix (Promega, M7122) and 200 nM primers was used for various fragment amplifications. Primer sequences are shown in Supplementary Table 7. A touchdown PCR protocol was used as follows: initial denaturation at 94 °C for 30 s; annealing at 62 °C for 30 s, decreasing by 0.5 °C per cycle; and extension at 72 °C for 60 s, followed by repeating these steps for eight cycles. After enrichment, the program continued for 29 cycles as follows: 94 °C for 30 s, 58 °C for 30 s, and 72 °C for 60 s. A 20 μl PCR with Phusion High-Fidelity DNA Polymerase (New England Biolabs, M0530) was performed to verify the *Yr27* gene sequence and to clone the silencing fragments following the manufacturer’s instructions. A 5 μl reaction (2.5 μl of KASP Master Mix (Low ROX KBS-1016-016), 0.07 μl of assay mix and 2.5 μl (25 ng) of DNA) was used for KASP markers. PCR cycling was performed in an ABI QuantStudio 6 Flex Real-Time PCR machine as follows: preread at 30 °C for 60 s; hold stage at 94 °C for 15 min; and then ten touchdown cycles (94 °C for 20 s; touchdown at 61 °C, decreasing by 0.6 °C per cycle for 60 s), followed by 29 additional cycles (94 °C for 20 s; 55 °C for 60 s). The plates were then read at 30 °C for endpoint fluorescent measurement.

### Candidate gene identification

To identify the candidate gene for *QYr.sgi-2B.1*, we anchored the flanking markers AX-94723778 and IWA4472 to the Kariega pseudomolecules. Annotated high-confidence genes and putative NLR loci predicted by NLR-Annotator^55^ at the delimited physical interval were selected as putative candidate genes. NLRs predicted at the target interval were prioritized for further analysis. First, the Kariega NLR sequences were screened for homology using the Basic Local Alignment Search Tool (BLAST) to identify the sequence diversity between Kariega and other wheat cultivars. Second, exon sequences of the shortlisted NLRs were amplified from Kariega, Avocet 2B and the ten susceptible EMS mutants. The amplicons were Sanger sequenced to identify and verify EMS mutations. Primers 2BNLR5F3/R8 and 2BNLR5F10/R12 were used to amplify exon sequences of *QYr.sgi-2B.1* (Supplementary Table 7).

### VIGS

To develop the VIGS probes, the predicted *QYr.sgi-2B.1* coding sequence was searched against the Kariega whole transcriptome (RNA-Seq and Iso-Seq) database using siRNA-Finder (si-Fi) software^59^. Based on the RNA interference (RNAi) design plot, three regions (Si1 (197 bp) in the 5′ UTR, Si2 (200 bp), and Si3 (198 bp) in the coding sequence) were selected for VIGS. The selected probes were verified for specificity using a BLAST search against the Kariega genome assembly (<80% sequence identity for hits other than *Yr27*). The target sequences were cloned into the pBS-BSMV-γ (BSMV, barley stripe mosaic virus) vector in an antisense direction^60,61^. Viral RNAs of BSMV–GFP and BSMV–GUS were used as controls, along with the BSMV–Si1, BSMV–Si2 and BSMV–Si3 constructs to infect the plants. In brief, *Agrobacterium tumefaciens* strain GV3101 carrying the viral pBS-BSMV-α, pBS-BSMV-β and pBS-BSMV-γ plasmids, with the γ plasmid carrying the VIGS target sequence, was infiltrated into *Nicotiana benthamiana*. Infiltrated leaves were harvested 4 days after inoculation and homogenized with virus inoculation buffer. The leaf extracts were used to infect 15-day-old wheat plants. Then, seedlings were placed in a growth chamber (60% humidity, 16/8 h light/dark regime with temperature set to 21/18 °C). At the flag leaf stage (approximately 20 days after viral infection), stripe rust inoculation was carried out, and the flag leaves were phenotyped 15 days after inoculation.

### Quantitative real-time PCR

Leaf materials inoculated with stripe rust were collected at different time points in five biological replicates for RNA isolation. RNA extraction was done using the Maxwell RSC Plant RNA kit and the Maxwell RSC48 instrument (Promega). One microgram of RNA was used for the first-strand cDNA synthesis using a High-Capacity cDNA Reverse Transcription Kit following the user guidelines (Applied Biosystems, 4368814). cDNA was further diluted 20-fold, and 4 μl of diluted cDNA were used for qPCR (quantitative real-time PCR). qPCR was performed using the 2BNLR5F17/R11 primers (Supplementary Table 7). A 20 μl qPCR was set up and run on the ABI QuantStudio 6 Flex Real-Time PCR machine using PowerUp SYBR Green Master Mix (Applied Biosystems, AS25741). The $2^{{-\Delta}{\Delta}{Ct}}$ method was used to normalize transcript values relative to endogenous controls Ta.6863 (ref. ^62^).

### Haplotype analysis, Shannon entropy and homology modeling

Yr27 protein versions were retrieved from the National Center for Biotechnology Information (NCBI) using a protein BLAST search (Supplementary Table 8) and aligned using MUSCLE in Geneious Prime 2019. Haplotypes were classified based on amino acid variations (Extended Data Fig. 5a). To identify residues overlapping functional motifs, the Motif Alignment Search Tool (MAST v.5.3.3)^63^ was used to predict the predefined NLR motifs^64^ and LRRpredictor was used to predict canonical LXXLXLXX repeats^65^. Shannon entropy values were calculated using the Entropy-one tool in the HIV sequence database (https://www.hiv.lanl.gov/content/sequence/ENTROPY/entropy_one.html). The raw output values were plotted using GraphPad Prism v.9.2.0. The Yr27 amino acid sequence was submitted to the Protein Homology/analogY Recognition Engine v.2.0 (Phyre2) for homology modeling to predict the three-dimensional structure^66^. The resulting top two Yr27 models comprise residues D87 to L1060 (90%) aligned to RPP1 with 18% identity and 100% confidence, and residues P43 to I977 (87%) aligned to ZAR1 with 22% identity and 100% confidence. The coordinates of RPP1-CJID-ATR1 and ZAR1-RKS1-PBL2 cryo-electron microscopy (cryo-EM) structures were retrieved from the Protein Data Bank (PDB) (7CRC^27^ and 6J5T^67^) for further analysis. Based on the structure analysis, graphical illustrations were created in UCSF ChimeraX v.1.3dev2021 software^68^ using RPP1-CJID-ATR1 as reference.

### Reporting Summary

Further information on research design is available in the Nature Research Reporting Summary linked to this article.

## Online content

Any methods, additional references, Nature Research reporting summaries, source data, extended data, supplementary information, acknowledgements, peer review information; details of author contributions and competing interests; and statements of data and code availability are available at 10.1038/s41588-022-01022-1.

## Supplementary information

Supplementary Information Supplementary Tables 1–8.

Reporting Summary

Peer Review Information

## Data Availability

Data supporting the findings of this work are available within the paper and its Supplementary Information. The raw sequencing data used for de novo whole-genome assembly, CCS reads, raw bionano map, Omni-C reads, Kariega genome assembly, RNA-Seq data and Iso-Seq data for the annotation are available on the European Bioinformatics Institute (EBI) European Nucleotide Archive (ENA) under study number PRJEB45541. The *Yr27* coding sequence and genomic sequences have been deposited in the ENA under accession numbers OU248057 and OU248342, respectively. The whole-genome assembly and annotations of gene models, repeats and NLRs are available on the DRYAD database (10.5061/dryad.nk98sf7td).
