# Supplementary material for: Long-read genome sequencing of bread wheat facilitates disease resistance gene cloning
Source: Nat Genet. 2022 Mar 14;54(3):227–31. doi: 10.1038/s41588-022-01022-1 (PMC8920886; doi:10.1038/s41588-022-01022-1)
Supplement: Supplementary file 1 — Supplementary Tables 1–8. [file 41588_2022_1022_MOESM1_ESM.pdf]

nature portfolio

https://doi.org/10.1038/s41588-022-01022-1

Supplementary information

Long-read genome sequencing of bread wheat facilitates disease resistance gene cloning

In the format provided by the authors and unedited

Supplementary table 1. Details for each of the 20 PacBio SMRT cells.

Name	ENA sample ID	Number of reads	Total bases (>QV20)	Coverage (16 Gb)	Read length N50	Mean read quality	Median read quality
HiFi_1	ERS6590020	1,921,726	29,387,857,865	1.84	15,429	30.1	29.5
HiFi_2	ERS6590021	2,145,014	33,077,780,030	2.07	15,558	30.8	30.2
HiFi_3	ERS6590022	1,846,193	28,174,760,322	1.76	15,390	30.1	29.5
HiFi_4	ERS6590023	1,898,880	28,913,457,933	1.81	15,358	29.9	29.2
HiFi_5	ERS6590024	1,758,888	26,775,661,031	1.67	15,352	30.5	29.9
HiFi_6	ERS6590025	1,720,722	26,393,263,599	1.65	15,476	30.6	30.1
HiFi_7	ERS6590026	1,927,600	29,515,052,999	1.84	15,442	30.5	29.9
HiFi_8	ERS6590027	1,878,548	28,808,939,011	1.80	15,476	30.1	29.3
HiFi_9	ERS6590028	1,690,254	27,000,290,314	1.69	15,905	29.9	29.3
HiFi_10	ERS6590029	1,727,656	27,462,113,566	1.72	15,828	29.6	29
HiFi_11	ERS6590030	1,754,103	27,854,243,403	1.74	15,809	29.3	28.6
HiFi_12	ERS6590031	1,439,698	22,700,761,182	1.42	15,703	27.7	27.1
HiFi_13	ERS6590032	1,640,867	26,089,564,407	1.63	15,825	29.7	29.1
HiFi_14	ERS6590033	1,370,170	21,378,750,864	1.34	15,517	28.1	27.3
HiFi_15	ERS6590034	1,483,590	23,955,237,624	1.50	16,122	28.7	28
HiFi_16	ERS6590035	1,455,822	22,853,154,092	1.43	15,616	28.7	28
HiFi_17	ERS6590036	1,856,504	29,447,086,359	1.84	16,032	30.4	30
HiFi_18	ERS6590037	1,878,819	29,800,747,558	1.86	16,026	30.4	29.9
HiFi_19	ERS6590038	1,894,838	30,859,663,927	1.93	16,553	30	29.6
HiFi_20	ERS6590039	1,580,398	25,924,743,982	1.62	16,694	30.7	30.5

Supplementary table 2. Statistics of 20 individual Kariega assemblies with subsets of the total sequencing data. Shown are the results after assemblies with different numbers of SMRT cells. The lines highlighted in bold indicate the minimum coverage to reach a total assembly size >14 Gb (4 SMRT cells), contig N50 >1 Mb (5 SMRT cells), and contig N50 >10 Mb (8 SMRT cells).

Number of SMRT cells	Estimated coverage (16 Gb)	Number of contigs	Total assembly length	Min contig length	Max contig length	Contig N50	Contig N90
1	1.84	67,799	2,973,198,357	14,843	458,404	44,347	29,157
2	3.90	156,542	11,973,334,439	1,395	829,156	93,525	39,822
3	5.67	80,900	13,863,100,737	2,524	4,662,168	257,374	81,785
**4**	**7.47**	**33,368**	**14,337,789,701**	**1,805**	**5,938,990**	**755,994**	**223,966**
**5**	**9.15**	**16,223**	**14,480,828,885**	**3,464**	**14,115,195**	**1,939,342**	**530,046**
6	10.80	9,769	14,527,984,462	4,858	43,495,276	4,214,102	1,052,952
7	12.64	7,062	14,550,858,054	1,907	50,567,239	7,418,884	1,793,418
**8**	**14.44**	**6,085**	**14,570,886,426**	**1,899**	**82,520,403**	**10,926,647**	**2,440,285**
9	16.13	5,688	14,588,426,741	2,320	91,190,756	13,441,685	2,989,776
10	17.84	5,476	14,600,520,298	2,313	96,423,690	15,712,727	3,286,876
11	19.59	5,493	14,618,559,043	2,314	161,342,238	17,918,348	3,695,426
12	21.00	5,492	14,631,456,455	2,314	161,342,177	19,556,178	4,075,691
13	22.63	5,153	14,612,302,334	8,859	161,342,160	22,631,405	4,413,901
14	23.97	5,074	14,615,466,526	3,582	161,342,257	22,563,240	4,572,775
15	25.47	4,956	14,620,966,923	2,491	161,342,255	23,973,653	4,742,726
16	26.90	5,032	14,632,549,662	2,143	217,464,560	24,900,903	4,867,589
17	28.74	4,937	14,637,615,942	2,143	217,467,089	27,207,313	5,085,439
18	30.60	4,947	14,644,717,644	3,323	159,041,303	28,451,848	5,212,149
19	32.53	5,007	14,653,400,537	2,409	217,466,599	30,173,118	5,347,210
20	34.15	5,055	14,657,984,604	3,774	217,466,094	30,219,108	5,498,350

Supplementary table 3. Statistics of the Bionano optical map and hybrid assembly.

Molecules	
Filtered data (Gb)	2,880
Coverage (16 Gb)	180x
Molecule N50 (kb)	227
Average label density	13,7 / 100 kb
Optical map assembly	
Genome map count	968
Total genome map length (Gb)	14.3
Genome map N50 (Mb)	51.1
Alignment to contig assembly	
Effective coverage of assembly	125x
Average confidence	26
Hybrid scaffold assembly	
Total sequences	324
N50 length (Mb)	204.3
Max length (Mb)	627.2
Total length (Gb)	14.5
% of NGS in the hybrid scaffold	98,52
% of “N” bases in hybrid scaffolds	0,13
Gap number in hybrid scaffolds	2,771

Supplementary table 4. Number of assembled transcripts (RNA-Seq) for the six different tissues.

	RNA-Seq					
	**Seedling dusk**	**Flag leaf**	**Grain**	**Root**	**Seedling**	**Spike**
chr1A	5,085	5,040	6,166	6,793	3,768	6,749
chr1B	5,595	5,584	7,252	7,885	4,185	7,374
chr1D	4,894	4,859	6,065	6,480	3,750	6,395
chr2A	6,191	6,242	7,718	8,482	4,640	8,190
chr2B	6,840	6,895	8,405	9,697	4,948	8,947
chr2D	6,183	6,265	7,493	8,486	4,812	8,157
chr3A	5,858	5,981	7,237	7,578	4,364	7,804
chr3B	6,593	6,756	8,487	9,100	4,809	8,783
chr3D	5,663	5,870	7,095	7,685	4,301	7,567
chr4A	5,401	5,461	6,746	7,349	4,030	7,135
chr4B	5,120	5,065	6,513	7,029	3,780	6,534
chr4D	4,437	4,239	5,416	5,831	3,462	5,589
chr5A	5,950	5,817	7,258	7,927	4,420	7,757
chr5B	6,352	6,390	8,096	8,640	4,719	8,433
chr5D	5,984	5,909	7,576	7,931	4,749	7,619
chr6A	4,651	4,628	5,725	6,119	3,375	6,120
chr6B	5,539	5,712	7,444	7,722	4,339	7,478
chr6D	4,536	4,412	5,763	6,030	3,355	5,896
chr7A	5,814	5,783	7,154	7,835	4,166	7,734
chr7B	5,743	5,740	7,333	8,081	4,245	7,578
chr7D	5,784	5,684	7,260	7,975	4,313	7,624
chrUn	10,071	7,975	6,348	3,109	10,284	8,405
sum	128,284	126,307	154,550	163,764	98,814	163,868

Supplementary table 5. Number of assembled transcripts (Iso-Seq) for the six different tissues.

	Iso-Seq					
	Seedling dusk	Flag leaf	Grain	Root	Seedling	Spike
chr1A	3,894	3,513	799	3,702	2,184	2,906
chr1B	3,940	3,488	879	3,998	2,173	3,007
chr1D	3,622	3,336	823	3,682	2,142	2,913
chr2A	4,999	4,541	958	4,670	2,883	3,799
chr2B	5,042	4,582	974	4,833	2,904	3,789
chr2D	4,942	4,556	995	4,706	2,914	3,857
chr3A	4,484	4,186	816	4,152	2,595	3,370
chr3B	4,865	4,638	864	4,453	2,721	3,557
chr3D	4,453	4,280	830	4,331	2,682	3,472
chr4A	4,314	3,964	861	4,041	2,428	3,272
chr4B	4,160	3,655	794	3,873	2,284	2,999
chr4D	3,821	3,453	847	3,751	2,273	3,020
chr5A	4,402	3,981	888	4,099	2,548	3,356
chr5B	4,602	4,263	914	4,296	2,607	3,570
chr5D	4,459	4,244	949	4,355	2,635	3,520
chr6A	3,594	3,279	657	3,234	2,006	2,551
chr6B	4,613	4,039	721	3,667	2,471	2,967
chr6D	3,590	3,321	689	3,384	2,083	2,690
chr7A	4,442	4,140	824	3,948	2,424	3,270
chr7B	4,305	3,946	763	3,902	2,258	3,036
chr7D	4,254	4,088	857	4,064	2,404	3,374
chrUn	2,020	1,390	157	396	1,137	765
sum	92,817	84,883	17,859	85,537	52,756	69,060

Supplementary table 6. Genotyping of Kariega, Avocet S and Avocet 2B with the 35K Axiom® array.

**Introgression analysis**	
Total number of markers on array	32,498
Number of polymorphic markers between Kariega and Avocet S	4,813
Number of polymorphic markers showing Kariega allele in Avocet 2B	340

Chromosome locations of polymorphic markers that showed the Kariega allele in Avocet 2B*

**Chromosome**	**1**	**2**	**3**	**4**	**5**	**6**	**7**
**A**	0	8	6	1	0	2	1
**B**	1	**121**	23	2	1	0	1
**D**	0	18	9	1	2	2	0

*only reads of markers with unique hits in the Kariega genome were retained (199 markers).

Supplementary table 7. List of primers used in this study.

Marker	Sequence	Notes
Xbarc55	F: GCGGTCAACACACTCCACTCCTCTCTC<br>R: CGCTGCTCCCATTGCTCGCCGTTA	SSR marker
Xwmc344	F: ATTTCAGTCTAATTAGCGTTGG<br>R: AACAAAGAACATAATTAACCCC	SSR marker
IWB62112	FF: AAAAAGGGTGATTGCATTTGAATAT<br>HF: AAAAAGGGTGATTGCATTTGAATAC<br>R: TGTTGAAAATGAATGACTGTGAGA	KASP marker
IWB44515	FF: GAGATAAGCCTGCGTAATGTATTTT<br>HF: GAGATAAGCCTGCGTAATGTATTTC<br>R: CCGTTGTGTTTTCCAGGGC	KASP marker
IWB52095	FF: CGGTCATTCTTTCAGAAAGCATCTC<br>HF: CGGTCATTCTTTCAGAAAGCATCTA<br>R: GCTGTGCCGTATCATGCATC	KASP marker
IWB11751	FF: TGTGAACCAAACGTATCTTGTTATT<br>HF: TGTGAACCAAACGTATCTTGTTATC<br>R: AACTCAGAACCAGGCCTGTA	KASP marker
IWA4472	FF: GCTCGCTTTCATCATCGCT<br>HF: GCTCGCTTTCATCATCGCC<br>R: CAGTGGCAGGTTGTTGGC	KASP marker
AX-94723778	FF: AGCTCCTGGAAGACAAGGAC<br>HF: AACCTTCCATCCCTGGAGG<br>R: ATGCAAGGCGGTAGCAAT	KASP marker
KASPYr27	FF: AACCTTCCATCCCTGGAGA<br>HF: ACCTTCCATCCCTGGAGG<br>R: ATGCAAGGCGGTAGCAAT	*Yr27* gene specific KASP marker
Yr27Si1	F: AAGGAAGTTTAACGAATGCTTCTTGTTCGCG<br>R: AACCACCACCACCGTGGCCCTCGTGTTCTTCCAA	Amplification of Si1 in antisense
Yr27Si2	F: AAGGAAGTTTAATACTAGGTGCTCCAACCTCAA<br>R: AACCACCACCACCGTCGGCACCCTACCTTCAACG	Amplification of Si2 in antisense
Yr27Si3	F: AAGGAAGTTTAAGACAATATTCTCCAGTGGTGGC<br>R: AACCACCACCACCGTCTCAGCCCTTGCTAAGATAGAAT	Amplification of Si2 in antisense
2BNLR5F3	CCTAATGCATCCCTTGTGCTTGCGGTG	2BNLR5F3/R8: exon 3 amplification
2BNLR5F4	AGGTAAAAGCTGAAGGCGTAAGAG	Internal sequencing primer
2BNLR5F5	ACGTTATGCACGGAACATTCCACGG	Internal sequencing primer
2BNLR5F6	CTATAGACAGTGATGAGCCCAATA	Internal sequencing primer
2BNLR5F7	GATGATGGGGCGGGATGGAGGGAG	Internal sequencing primer
2BNLR5F8	CTGCATAAGCTCAGAATTATAGAGC	Internal sequencing primer
2BNLR5F10	GTGGGAACAGGCGACTCGAAGACGT	2BNLR5F10/R12: exon 4 amplification
2BNLR5F11	ATGGCCATGATCTTACCAGC	2BNLR5F11/R11: full CDS
2BNLR5F17	TTAGCATTACGGGCTGTCCAA	2BNLR5F17/R11 RT-qPCR primer
2BNLR5R4	CCGCGCTCGAGTGGCTGTTGTATCT	Internal sequencing primer
2BNLR5R5	GTGCATTATCGCGTGACTCCTCTG	Internal sequencing primer
2BNLR5R6	GACTCTATCTATGTCACGCGGA	Internal sequencing primer
2BNLR5R7	ATTCTGTCATTCTTGCATTAC	Internal sequencing primer
2BNLR5R8	AAATAATGACAACGGACGTTTGGT	Internal sequencing primer
2BNLR5R11	TCGTCCACTTGAATTAATCAGC	Reverse primer for RT-qPCR
2BNLR5R12	ATCCGGTGTTCTTAGTGGCA	Internal sequencing primer

FF: FAM (GAAGGTGACCAAGTTCATGCT) HF: HEX (GAAGGTCGGAGTCAACGGATT)

Supplementary Table 8. List of Yr27 haplotypes used in this study.

Accession	Source	Haplotype
Kariega	TraesKAR2B01G0121530LC	H1 (Yr27)
Kauz	this study	
Kubsa	this study	
Opata 85	this study	
Selkirk	this study	
Yr27/6*Avocet S	this study	
Avocet R	QUJ24313.1	H2
Aztec	QUM93369.1	(Lr13_ha)
Claire	Grain genes Blast	
Fuyu3	QUM93356.1	
Jagger	TraesJAG2B01G199800	
JC1016	QUM93364.1	
Jimai19	QUM93362.1	
Kabka3	QUM93368.1	
KF3	QUM93363.1	
CDC Landmark	TraesLDM2B01G194200	
LrZH22	QQY97227.1	
M114	QUM93357.1	
Mace	TraesMAC2B01G203300	
Manitou	QUM93365.1	
Nongda212	QUM93359.1	
Shidong7	QUM93358.1	
SY Mattis	TraesSYM2B01G197700	
Tanori71	QUM93367.1	
WL711	GEWU01168036.1	
Yan2415	QUM93361.1	
Yannong15	QUM93360.1	
Zhengnong17	QWY04414.1	
Zhou8425B	QUM93355.1	
Kronos	Grain genes Blast	
RL4013	QUM93366.1	
Zhoumai22	QUM93354.1	
Blackhull	QWY04417.1	H3
Cadenza	QUJ24314.1	

Weebil	GCA_902810675.1	H4
Julius	TraesJUL2B01G200000	(Lr13_hb)
Vakka	QWY04418.1	H5
Fielder	QUM93379.1	H6
Kenong213	QUM93381.1	H7
Paragon	GCA_902810665.1	
Mingxian169	QUM93380.1	H8
Yangbaimai	QWY04419.1	
Kechengmai	QUM93378.1	H9
Huaimai21	QUM93387.1	
Honglouguding	QUM93377.1	
Aikang58	QUM93375.1	H10
Svevo	TRITD2Bv1G060290	
Xindong31	QUM93385.1	
W301	QUM93384.1	
Norin 61	TraesNOR2B01G204600	H11
Yumai14	QWY04415.1	
Zimai1	QUM93376.1	
Xinshuguan	QUM93386.1	H12
Zhongmai415	QUM93382.1	H13
ArinaLrFor	TraesARI2B01G201600	H14
Zavitan	TRIDC2BG023690	H15
Robigus	GCA_902810685.1	H16
Jinhuanmai	QUM93383.1	H17
Chinese Spring	TraesCS2B02G182800	H18
Songruimai	QUM93371.1	
Boerqing	QUM93372.1	
PI190962 (spelt)	TraesTSP2B01G201300	H19
Yangmai12	QUM93374.1	H20
Zhengzhou5389	QUM93373.1	
