# Supplementary material for: Long-read genome sequencing of bread wheat facilitates disease resistance gene cloning
Source: Nat Genet. 2022 Mar 14;54(3):227–31. doi: 10.1038/s41588-022-01022-1 (PMC8920886; doi:10.1038/s41588-022-01022-1)
Supplement: Supplementary file 2 — Reporting Summary [file 41588_2022_1022_MOESM2_ESM.pdf]

nature portfolio

Corresponding author(s): Simon Krattinger

Last updated by author(s): Jan 16, 2022

Reporting Summary

Nature Portfolio wishes to improve the reproducibility of the work that we publish. This form provides structure for consistency and transparency in reporting. For further information on Nature Portfolio policies, see our Editorial Policies and the Editorial Policy Checklist.

Statistics

For all statistical analyses, confirm that the following items are present in the figure legend, table legend, main text, or Methods section.

n/a	Confirmed	
☐	☒	The exact sample size (*n*) for each experimental group/condition, given as a discrete number and unit of measurement
☐	☒	A statement on whether measurements were taken from distinct samples or whether the same sample was measured repeatedly
☐	☒	The statistical test(s) used AND whether they are one- or two-sided<br>*Only common tests should be described solely by name; describe more complex techniques in the Methods section.*
☒	☐	A description of all covariates tested
☒	☐	A description of any assumptions or corrections, such as tests of normality and adjustment for multiple comparisons
☐	☒	A full description of the statistical parameters including central tendency (e.g. means) or other basic estimates (e.g. regression coefficient) AND variation (e.g. standard deviation) or associated estimates of uncertainty (e.g. confidence intervals)
☐	☒	For null hypothesis testing, the test statistic (e.g. *F*, *t*, *r*) with confidence intervals, effect sizes, degrees of freedom and *P* value noted<br>*Give P values as exact values whenever suitable.*
☒	☐	For Bayesian analysis, information on the choice of priors and Markov chain Monte Carlo settings
☒	☐	For hierarchical and complex designs, identification of the appropriate level for tests and full reporting of outcomes
☒	☐	Estimates of effect sizes (e.g. Cohen's *d*, Pearson's *r*), indicating how they were calculated

Our web collection on statistics for biologists contains articles on many of the points above.

Software and code

Policy information about availability of computer code

Data collection	Other plant protein sequences were downloaded from the UniProt/SwissProt database (Release 2021_03 – [https://www.uniprot.org/]) and the Poales _odb10 BUSCO dataset [https://busco.ezlab.org/frames/plants.htm].<br>The TREP database was downloaded from [https://botserv2.uzh.ch/kelldata/trep-db/].<br>KASP primer sequences for the 90K iSelect SNPs were downloaded from the CerealsDB database [https://www.cerealsdb.uk.net/cerealgenomics/CerealsDB/KASP_primers_for_iSelect.php].<br>NCBI blastp suite was used to retrieve the protein sequences of Yr27 haplotypes [https://blast.ncbi.nlm.nih.gov/].<br>The cryo-EM structures of reference proteins were retrieved from Protein Data Bank [https://www.rcsb.org/].
Data analysis	HifiAsm v0.11 was used for the whole genome assembly.<br>Bionano Solve 3.6 was used for the hybrid-scaffolding.<br>Juicer tools (v1.6), 3D-DNA (v180114) and Juicebox (v1.11.08) were used for the pseudomolecule construction.<br>MashMap (v.2.0) was used for the genome alignment analysis.<br>IsoSeq pipeline (v3 - https://github.com/PacificBiosciences/IsoSeq), STAR (v.2.7.0f), Stringtie (v2.1.4), minimap2 (v2.17-r941) and cDNA_Cupcake (https://github.com/Magdoll/cDNA_Cupcake) were used for the transcript and isoform assemblies.<br>Transdecoder (v5.5.0), GenomeThreader (v1.7.1), Augustus (v3.4.0), GeneMark (v4.38), FgeneSH (v8.0.0), EDTA (v1.9.4), EVidenceModeler (v1.1.1), PASA (v2.4.1), DIAMOND (v2.0.9) and NLR-annotator were used for the gene, repeat and NLR annotations.<br>MapDisto (v2.0) and MapChart (v2.3.2) were used to generate genetic maps<br>si-Fi21_1.2.3-0008 was used to predict the probes for virus-induced gene silencing.<br>Geneious prime 2019.2.3 was used for various sequence alignment and analysis<br>Phyre (v2.0) was used to generate the homology models of Yr27 and Lr13 (http://www.sbg.bio.ic.ac.uk/phyre2/html/page.cgi?id=index)<br>Entropy-One tool listed in the HIV sequence database was used to calculate the Shannon entropy score (https://www.hiv.lanl.gov/content/sequence/ENTROPY/entropy_one.html)<br>MUSCLE (v3.8.425) was used to align the protein sequences<br>MAST (v5.3.3) was used to predict the NLR motifs (https://meme-suite.org/meme/meme_5.3.3/tools/mast)

LRRpredictor (v1.0) was used to predict the Leucine rich repeats (https://lrrpredictor.biochim.ro/)
GraphPad Prism (v9.2.0) was used to plot graphs
UCSFChimeraX (v1.3dev2021) software was used to analyze the protein models and generate graphical illustrations

For manuscripts utilizing custom algorithms or software that are central to the research but not yet described in published literature, software must be made available to editors and reviewers. We strongly encourage code deposition in a community repository (e.g. GitHub). See the Nature Portfolio guidelines for submitting code & software for further information.

Data

Policy information about availability of data

All manuscripts must include a data availability statement. This statement should provide the following information, where applicable:

- Accession codes, unique identifiers, or web links for publicly available datasets
- A description of any restrictions on data availability
- For clinical datasets or third party data, please ensure that the statement adheres to our policy

Protein coding sequences of the 10+ wheat genomes project were downloaded from Ensembl Plants [https://plants.ensembl.org/index.html] and for the Chinese Spring accession from [https://wheat-urgi.versailles.inra.fr/Seq-Repository/Assemblies].
The raw sequencing data used for de novo whole-genome assembly, the raw bionano map, the Kariega genome assembly, the transcriptomics data (RNA-Seq and Iso-Seq) and the specific CDS and genomic sequences of Yr27 are available on EBI-ENA under the study number PRJEB45541. The Yr27 CDS and genomic sequences have been deposited in ENA under accession numbers OU248057 and OU248342, respectively.
The genome assembly and gene, repeat and NLR annotations are available on the DRYAD database under [https://doi.org/10.5061/dryad.nk98sf7td

Field-specific reporting

Please select the one below that is the best fit for your research. If you are not sure, read the appropriate sections before making your selection.

☒ Life sciences ☐ Behavioural & social sciences ☐ Ecological, evolutionary & environmental sciences

For a reference copy of the document with all sections, see nature.com/documents/nr-reporting-summary-flat.pdf

Life sciences study design

All studies must disclose on these points even when the disclosure is negative.

Sample size	Sample sizes are described in the manuscript. Size of Avocet 2B x Avocet S F2 population = 345 plants. Size of Avocet 2B mutant population = ~2,000. No statistical method was used to predetermine sample size. Sample sizes have been chosen based on commonly reported sample sizes in literature.
Data exclusions	No data were excluded.
Replication	The phenotypes of the ten mutants (identified in the M1 generation) were confirmed in the M2 and M3 generations. Phenotyping at M3 generations was repeated twice independently with successful sporulation in negative controls to validate the experiment. The silencing experiment was performed independently twice with five biological replications per construct per experiment. Successful rust phenotype on appropriate controls were used to validate both experiments. RT-qPCR was repeated twice with three to five biological and three technical replicates.
Randomization	Inoculated plants were allocated randomly among groups.
Blinding	Blinding was performed when phenotyping plants (i.e., the genotype of the plant was not known when the phenotypic data were recorded)

Reporting for specific materials, systems and methods

We require information from authors about some types of materials, experimental systems and methods used in many studies. Here, indicate whether each material, system or method listed is relevant to your study. If you are not sure if a list item applies to your research, read the appropriate section before selecting a response.

Materials & experimental systems

n/a	Involved in the study	
☒	☐	Antibodies
☒	☐	Eukaryotic cell lines
☒	☐	Palaeontology and archaeology
☒	☐	Animals and other organisms
☒	☐	Human research participants
☒	☐	Clinical data
☒	☐	Dual use research of concern

Methods

n/a	Involved in the study	
☒	☐	ChIP-seq
☒	☐	Flow cytometry
☒	☐	MRI-based neuroimaging
