# Supplementary material for: Long-read genome sequencing of bread wheat facilitates disease resistance gene cloning
Source: Nat Genet. 2022 Mar 14;54(3):227–31. doi: 10.1038/s41588-022-01022-1 (PMC8920886; doi:10.1038/s41588-022-01022-1)
Supplement: Supplementary file 3 — Peer Review Information [file 41588_2022_1022_MOESM3_ESM.pdf]

natureresearch

# Peer Review Information

**Journal:** Nature Genetics
**Manuscript Title:** Long-read genome sequencing of bread wheat facilitates disease resistance gene cloning
**Corresponding author name(s):** Professor Simon Krattinger

## Reviewer Comments & Decisions:

**Decision Letter, initial version:**

10th Aug 2021

Dear Professor Krattinger,

Your Letter, "Long-read bread wheat genome enables disease resistance gene cloning" has now been seen by 4 referees. You will see from their comments copied below that while they find your work of considerable potential interest, they have raised quite substantial concerns that must be addressed. In light of these comments, we cannot accept the manuscript for publication, but would be very

interested in considering a substantially revised version that fully addresses these serious concerns.

We hope you will find the referees' comments useful as you decide how to proceed. If you wish to submit a substantially revised manuscript, please bear in mind that we will be reluctant to approach the referees again in the absence of major revisions.

As you will see from these comments, reviewer #1 notes that the study contains two independent parts (genome sequencing and cloning of disease resistance gene Yr27) but neither of them is groundbreaking. This reviewer has serious concerns regarding the genome assembly and comparisons with other wheat genomes, as well as a few limitations in the cloning of Yr27 that need to be improved. Reviewer #2 thinks that the identification of an NBS gene Yr27 is not conceptually novel, and that some of the conclusions are overstated and speculative. Reviewers #3-4 provide overall positive assessments, though have concerns about the novelty and suggestions for extending the functional work on the identified gene Yr27. Please address all referees' points as thoroughly as possible.

If you choose to revise your manuscript taking into account all reviewer and editor comments, please highlight all changes in the manuscript text file. At this stage we will need you to upload a copy of the manuscript in MS Word .docx or similar editable format.

We are committed to providing a fair and constructive peer-review process. Do not hesitate to contact us if there are specific requests from the reviewers that you believe are technically impossible or unlikely to yield a meaningful outcome.

If revising your manuscript:

*1) Include a "Response to referees" document detailing, point-by-point, how you addressed each referee comment. If no action was taken to address a point, you must provide a compelling argument. This response will be sent back to the referees along with the revised manuscript.

*2) If you have not done so already please begin to revise your manuscript so that it conforms to our Letter format instructions, available <a href="http://www.nature.com/ng/authors/article_types/index.html">here</a>.
Refer also to any guidelines provided in this letter.

*3) Include a revised version of any required Reporting Summary:
https://www.nature.com/documents/nr-reporting-summary.pdf
It will be available to referees (and, potentially, statisticians) to aid in their evaluation if the manuscript goes back for peer review.
A revised checklist is essential for re-review of the paper.

Please be aware of our <a href="https://www.nature.com/nature-research/editorial-policies/image-integrity">guidelines on digital image standards.</a>

You may use the link below to submit your revised manuscript and related files:

[REDACTED]

<strong>Note:</strong> This URL links to your confidential home page and associated information about manuscripts you may have submitted, or that you are reviewing for us. If you wish to forward this email to co-authors, please delete the link to your homepage.

If you wish to submit a suitably revised manuscript we would hope to receive it within 6 months. If you cannot send it within this time, please let us know. We will be happy to consider your revision so long as nothing similar has been accepted for publication at Nature Genetics or published elsewhere. Should your manuscript be substantially delayed without notifying us in advance and your article is eventually published, the received date would be that of the revised, not the original, version.

Please do not hesitate to contact me if you have any questions or would like to discuss the required revisions further.

Nature Genetics is committed to improving transparency in authorship. As part of our efforts in this direction, we are now requesting that all authors identified as 'corresponding author' on published papers create and link their Open Researcher and Contributor Identifier (ORCID) with their account on the Manuscript Tracking System (MTS), prior to acceptance. ORCID helps the scientific community achieve unambiguous attribution of all scholarly contributions. You can create and link your ORCID from the home page of the MTS by clicking on 'Modify my Springer Nature account'. For more information please visit please visit <a href="http://www.springernature.com/orcid">www.springernature.com/orcid</a>.

Thank you for the opportunity to review your work.

Sincerely,

Wei

Wei Li, PhD
Senior Editor
Nature Genetics
New York, NY 10004, USA
www.nature.com/ng

Reviewers' Comments:

Reviewer #1:
Remarks to the Author:
This work illustrates how using long read based genome sequencing is possible to clone a resistant gene in wheat. The paper is made of two rather independent parts, genome sequencing and R gene cloning but none of them is really a breakthrough in science. In current days, genome sequencing is only a matter of technology and it is well known that PacBio Hi-Fi outperform previously sequencing methods. The cloned R gene is a novel allele of an already known gene and does not uncover any novel resistant mechanisms.

The latest technologies have been applied to sequence the wheat genome and to assembly a high-quality genome. The quality level of the assembly is definitely high but within the expectation of the technologies applied. Looking the paper from a sequencing point of view, the gene annotation made with only 6 tissues is below standard (curiously no rust infected tissues have been used) and there are no comparative data of Kariega with all others wheat genome sequenced so far. Overall, I suppose that genome sequence is not the focus of this manuscripts. In Table 1 BUSCO, is indicated "duplicated

96.3%", duplicated means exactly 3 copies?
The cloning of Yr27, a novel allele of Lr13, is an interesting result, but there are some weaknesses that the authors should address.
In figure 1 panel A Kariega and Avocet S show full susceptibility to rust at seedlings stage suggesting that the resistance loci carried by Kariega are effective only in adult plant. Avocet 2B derived from Avocet S after the introgressed the QYr.sgi-2B.1 from Kariega. As reported in Figure1 panel A and indicated in the text, Avocet 2B shows a moderate seedling resistance and the leaves presented in figure 1A have some green sectors that contrast with the leaves of Kariega and Avocet S fully covered with rust. The leaves presented in Supplementary Figure S3 (assuming that they represent leaves after inoculation, the legend does not describe the second part of this figure) still confirm the moderate resistance of Avocet 2B at seedling stage.
Overall, these data suggest that that Avocet 2B expresses a trait that is neither expressed in Avocet S nor in Kariega. The moderate resistance of Avocet 2B is not expected and open several questions. Why the resistant locus (Yr27) in Avocet express some resistance also in seedling while in Kariega (with 3 R genes) no effect was detected? Are the authors sure that Avocet 2B carry ONLY the QYr.sgi-2B.1 introgression? How was this introgression assessed? In the contest of this paper, a whole genome sequencing could have been used to assure that no other interfering regions have been introgressed in Avocet 2B.
In figure 1A flag leaves, the leaf representing Avocet 2B show some level of disease, possibly right assuming that the full resistant phenotype of Kariega is due to 3 R genes. Nevertheless, in panel 1D the same sample (Avocet 2B) is completely green and no symptoms are present, Avocet 2B in panel 1A-flag leaves and in panel 1D have the same name but they look quite different. Notably, some of the mutants presented in Figure 1D, although clearly different from 1D control, are not so different from Avocet 2B presented in panel 1A-flag leaf. Which control is right?
Considering the unexpected (or unexplained) moderate resistance phenotype expressed by Avoced 2B at seedling stage a gain of function experiment of Yr27 and Avocet S should have been carried out to complement the loss of function experiments and to prove if the seeding resistance is due to Yr27 in the Avocet background of to other genetic factors.
In Supplementary figure S3, the sample "Avocet 2B 24 hrs of inoculation has an incredible big error bar that makes a relative expression value of 4 not significantly different from the value of time zero (about 1). This experiment could be repeated.

Reviewer #2:
Remarks to the Author:
A. A high quality genome assembly was created for the wheat cultivar Kariega, which has durable resistance to yellow rust. The durable resistance is the result of stacking of multiple resistance genes in the line. The QTL, which is responsible for some resistance, QYr.sgi-4A.1 was isolated in a recombinant population and found to provide resistance to one rust isolate (6E22A+), so the gene only accounts for one race in the durable resistance profile. The candidate gene was provided by homology of several genes to NBS type resistance genes. EMS mutagenesis of a resistant line (Avocet B) resulted in ten mutants at one of the NBS genes, which was labeled Yr27. The gene is expressed, and expression is elevated upon inoculation in seedlings. Silencing of the gene also resulted in susceptible plants. The authors add the added finding that the gene is closely related to other resistance genes and extrapolate that small changes in coding of Yr27 should provide resistance against other pathogens.

B. The findings are significant in the dissection of durable resistance and a high quality map of hexaploid wheat. The identification of an NBS gene as a resistance gene is not novel but the work was well-done and constitutes considerable effort in the identification of the gene. The gene was not transferred to a susceptible line by gene transfer. Nonetheless, a variety of techniques were used to

corroborate the identify of Yr27.

C. The data and methodology was well presented.

D. The use of statistics was appropriate.

E. The Results were straightforward and robust.

F. The paper would be extraordinary, if manipulation of the gene led to new resistance specificity rather than just postulated.

Some minor comments:
1. "private to Yr27" is regional use of English (line 109)
2. Why are amino acid stretches approximate (eg line 104)
3. Avoid injecting assumptions, eg "surpisingly" (line 96)

G. The references were adequate.

H. The paper was clear. However, the conclusion that the results "show that highly similar alleles of single-copy genes can confer resistance to different pathogen, providing a basis for engineering immune receptors with multiple recognition specificities in future" contains a number of assumptions not supported by research and is largely speculative. The thought could be stated prior to the research given the wide range of NBS and RLK homologs with different specificities.

Reviewer #3:
Remarks to the Author:
The authors took the advantage of recently fast developing field in sequencing and bioinformatic analysis and cloned a gene Yr27 conferring stripe rust resistance in wheat. Interestingly, they were able to show that Yr27 and recently cloned Lr13 were allelic, however, conferred resistance to different pathogens. The manuscript was very clearly and well written and I enjoyed reading it. Even though the gene structure is not something novel, the manuscript presented a breakthrough in combining different techniques to get a good genome assembly within very short period of time for wheat, which has a large genome size of about 16Gb. Their manuscript also provided useful information on the techniques so that the other researchers working on wheat and other plant species can all benefit from.

After identifying the gene candidate with mutants, VIGS was used to confirm the candidate. Although stable transformation would be even better, the evidence provided in the manuscript was solid enough to validate the candidate.

Suppl. Fig. 3: Top panel, 35 day-old plants, there is a hike at 24hpi for Avocet 2B, which is very different compared to the rest. Any explanation?
Is it correct that 35 day-old plants are already at flag-leaf stage?
For 35 day-old plants, the rust response for Avocet S does not seem right and is too low, which should be highly susceptible. Any explanation?

There are some addtional points for the authors to attend to:
L40: There are current 83 catalogued Yr genes, instead of 78, please revise;
The Yr genes cloned: Yr5 (Yr5a), YrSP (Yr5b), Yr7, Yr15, Yr36; Yr18 and Yr46 in the complex; and three temporary designated genes Yr10cg, YrAS2388R and YrU1.

L115: For the ~11-fold coverage, the N50 is close to 5.0 Mb instead of 4.2 Mb. Please check Fig. 2, the N50 curve.

Fig. 1g: "N895D" was actually magenta, not pink. Also change in the figure caption, from "highlighted" in brown or magenta to in brown or magenta font colour.

L197: Better write as "R:S = 254:91, $\chi 2$ = 0.348, …".

L328: Cat. No. M0880 is enough for EMS, package size is not necessary.

L375: Change to "Si3 (198bp) in CDS" to make it clearer.

Please update the references because several papers have been formally published and have page number now.

Suppl. Fig. 1: The green squares are not very visible, either change the colour or make the border thicker.

Suppl. Tables 4 and 5: In the title, "tissues" are more appropriate than "developmental stages".

Reviewer #4:
Remarks to the Author:
The authors demonstrate efficient cloning of disease resistance gene from wheat that was enabled by rapid construction of chromosome level genome assembly (14 Gb, polyploid) from long reads and other data. The study builds on breakthroughs in genome assembly methods and new and better sequencing technologies, yet it is not an incremental advance – the assembly authors present is many folds better than previous efforts and they demonstrate the feasibility and democratization of their approach surpassing the need to have international consortia. present a subsampling experiment to demonstrate assembly quality at different sequencing depth. Technically, the study is outstanding, and I have no further comments or concerns. The paper is very clearly written.

My main suggestion for authors is to provide more details on the identified gene Yr27. It is an NLR and there is nothing novel about it, yet there is a lot of underexplored biology in NLRs that can recognize different pathogens. Specifically, I would recommend illustrating natural diversity and critical variants using structural information for NB-ARC and LRR domain and place these amino acid changes in the functional context of the protein. Do amino acid polymorphisms in NB-ARC overlap with functional motifs? Do they cluster on the side of the domain that is in contact with the LRR and therefore controls receptor? Similarly, do LRR amino acid changes cluster on the concave side of the protein where a ligand would bind or are they likely in contact with NB-ARC? Is Yr27 a highly variable NLR based on its entropy footprint? A simple modeling with Phyre using newest available NLR structures can help to answer these questions and further enrich presented analyses.

**Author Rebuttal to Initial comments**

**Point-by-point responses**

Reviewer #1:
Remarks to the Author:
This work illustrates how using long read based genome sequencing is possible to clone a resistant gene in wheat. The paper is made of two rather independent parts, genome sequencing and R gene cloning but none of them is really a breakthrough in science. In current days, genome sequencing is only a matter of technology and it is well known that PacBio Hi-Fi outperform previously sequencing methods. The cloned R gene is a novel allele of an already known gene and does not uncover any novel resistant mechanisms.
Our response: *This manuscript demonstrates that it is now feasible, affordable, and attractive to generate high-quality assemblies of specific wheat cultivars for gene cloning. We see this manuscript as the latest chapter in a series of highly influential papers that described genomics-based gene cloning approaches for the large and complex wheat genome. Examples are MutRenSeq (Steuernagel et al. 2016, Nat Biotechnol 34:652-655), MutChromSeq (Sanchez-Martin et al. 2016, Genome Biol 17:221), TACCA (Thind et al. 2017, Nat Biotechnol 35:793-796) and AgRenSeq (Arora et al. 2019, Nat Biotechnol 37:139-143). In contrast to these earlier gene cloning protocols, the approach presented here eliminates the need for time-consuming genome complexity reduction (e.g. chromosome sorting or capture arrays), which will significantly speed up gene cloning in wheat. The genome sequencing and R gene cloning are thus tightly connected and complementary in our view. This is the first proof-of-principle study that demonstrates the usefulness of PacBio Hi-Fi for gene cloning in wheat. The discovery that the stripe rust resistance gene Yr27 is an allele of the leaf rust resistance gene Lr13 adds biological novelty in our view. We are not aware of any other example where two highly similar NLR alleles provide race-specific resistance against different pathogen species. In summary, we think that our work presents conceptual, technological, and biological novelty.*

The latest technologies have been applied to sequence the wheat genome and to assembly a high-quality genome. The quality level of the assembly is definitely high but within the expectation of the technologies applied. Looking the paper from a sequencing point of view, the gene annotation made with only 6 tissues is below standard (curiously no rust infected tissues have been used) and there are no comparative data of Kariega with all others wheat genome sequenced so far. Overall, I suppose that genome sequence is not the focus of this manuscripts.
Our response: *As stated above, a detailed comparative analysis was indeed not the focus of this manuscript. The number of tissues for gene model prediction is comparable to what has recently been used for other species where gene annotations were based on a combination of ab initio and evidence-based gene model predictions. Examples include the tea genome (6 tissues; Zhang et al. 2021, Nat Genet 53:1250-1259), the cotton genome (4 tissues; Ma et al. 2021, Nat Genet 53: 1385-1391) or the Pacific Island silvergrass (5 tissues; Zhang et al. 2021, Nat Plants 7:608-618). The number of predicted gene models in Kariega is comparable to previous wheat assemblies. The reason for not included rust infected tissues is that R genes are often not or only weakly induced by pathogen infections. We updated Supplementary Fig. 2 (now Extended Data Fig. 3) and made a comparison of the Kariega assembly with all wheat cultivars reported in the 10+ Wheat Genomes Project.*

In Table 1 BUSCO, is indicated “duplicated 96.3%”, duplicated means exactly 3 copies?

<u>Our response:</u> *Around 76% of the universal single-copy orthologs have exactly three copies. The remaining "duplicated" orthologs have two or more than three copies. We have prepared a new supplementary figure (Extended Data Fig. 2) that provides a detailed breakdown of the BUSCO analysis and compares Kariega to the 10+ Wheat Genomes Project assemblies. This comparison shows that the number of universal single-copy orthologs with two, three, or more copies are comparable across these assemblies.*

The cloning of Yr27, a novel allele of Lr13, is an interesting result, but there are some weaknesses that the authors should address.

In figure 1 panel A Kariega and Avocet S show full susceptibility to rust at seedlings stage suggesting that the resistance loci carried by Kariega are effective only in adult plant. Avocet 2B derived from Avocet S after the introgressed the QYr.sgi-2B.1 from Kariega. As reported in Figure1 panel A and indicated in the text, Avocet 2B shows a moderate seedling resistance and the leaves presented in figure 1A have some green sectors that contrast with the leaves of Kariega and Avocet S fully covered with rust. The leaves presented in Supplementary Figure S3 (assuming that they represent leaves after inoculation, the legend does not describe the second part of this figure) still confirm the moderate resistance of Avocet 2B at seedling stage. Overall, these data suggest that that Avocet 2B expresses a trait that is neither expressed in Avocet S nor in Kariega. The moderate resistance of Avocet 2B is not expected and open several questions. Why the resistant locus (Yr27) in Avocet express some resistance also in seedling while in Kariega (with 3 R genes) no effect was detected?
<u>Our response:</u> *This was very well observed. However, we do not think that these observations are unusual or unexpected. Yr27 is described as an intermediate seedling resistance gene and a dependence of the Yr27 phenotype on environment and genetic background is well established in literature. For example, the first description of Yr27 (Wellings et al. 1992 Vortr. Pflanzenzüchtg 24:273-275) reported that inoculations of Yr27 carrying wheat lines with the same stripe rust isolate produced different infection types in two different labs (environmental dependence) and on different wheat cultivars (genotype dependence). McDonald et al. (2004 Euphytica 136:239-248) state that "seedlings of wheat genotypes with Yr27 when infected with avirulent Australian Pst cultures produce necrotic low infection types with* **variable** *amounts of sporulation" and "while Yr27 was often difficult to score in segregating seedling populations, it is apparently quite effective in conferring resistance to avirulent cultures under field conditions". Similarly, the Wheat Rusts Atlas (https://globalrust.org/sites/default/files/wheat_rust_atlas_full.pdf) describes a range of resistance responses for Yr27 (known as Yr Selkirk in the Wheat Rusts Atlas). These observations indicate that the resistance response of Yr27 at seedling stage is influenced by genetic backgrounds and the environment. Genetic background dependences are quite common for rust resistance genes and might be due to the segregation of genetic modifiers. A similar observation, for example, was made for the leaf rust resistance gene Lr14a (Kolodziej et al. 2021, Nat Commun 12:956) where the backcross line ArinaLrFor used for gene cloning showed a stronger seedling resistance than either parent Arina and Forno. We have indicated the variable seedling response of Yr27 in the main text as follows (lines 98-101):'The phenotypic expression of Yr27 (previously known as YrSk) at seedling stage is influenced by the environment and the genetic background'.*

Are the authors sure that Avocet 2B carry ONLY the QYr.sgi-2B.1 introgression? How was this introgression assessed? In the contest of this paper, a whole genome sequencing could have been used to assure that no other interfering regions have been introgressed in Avocet 2B.
Our response: *Kariega, Avocet, and Avocet 2B were genotyped with the 35K Axiom® array, which confirmed that none of the additional stripe rust resistance QTL described in Kariega were introgressed into Avocet 2B. This was mentioned in the methods section. We have now included the SNP Array data in the new Supplementary Table 6. The stripe rust resistance in the 'Avocet 2B x Avocet S' population followed a single gene segregation, which provides additional proof that only one stripe rust resistance gene was introgressed into Avocet 2B.*

In figure 1A flag leaves, the leaf representing Avocet 2B show some level of disease, possibly right assuming that the full resistant phenotype of Kariega is due to 3 R genes. Nevertheless, in panel 1D the same sample (Avocet 2B) is completely green and no symptoms are present, Avocet 2B in panel 1A-flag leaves and in panel 1D have the same name but they look quite different. Notably, some of the mutants presented in Figure 1D, although clearly different from 1D control, are not so different from Avocet 2B presented in panel 1A-flag leaf. Which control is right?
Our response: *Both controls are right. As stated above, the level of the resistance response depends on the growth conditions of the plants, the inoculum load and the genetic background. The Avocet 2B shown in Fig. 1a were inoculated in a greenhouse in South Africa while the Avocet 2B shown in Fig. 1d were grown in a growth room at KAUST. Such slight differences in disease responses are not unusual for rust resistance genes in general and for Yr27 in particular. Most importantly, the appropriate controls (Avocet S and Avocet 2B) were included in all the experiments to allow for a direct comparison of the results. For clarification, we have now also included the appropriate Avocet S control in Fig. 1d (i.e., the Avocet S plants that were grown and inoculated together with this mutant batch).*

Considering the unexpected (or unexplained) moderate resistance phenotype expressed by Avoced 2B at seedling stage a gain of function experiment of Yr27 and Avocet S should have been carried out to complement the loss of function experiments and to prove if the seeding resistance is due to Yr27 in the Avocet background of to other genetic factors.
Our response: *Given the above references describing a 'background dependence' of Yr27 we do not think that the differences in seedling phenotype between Kariega and Avocet 2B are unexpected. The genetic definition of a resistance gene provided by Sanchez-Martin and Keller (2021, Current Opinion in Plant Biology 62:10253) states that "the term 'resistance gene' has been used to describe the polymorphic genetic component that makes the crucial difference between resistant and susceptible genotypes". According to this definition, Yr27 is the only resistance gene in Avocet 2B because (i) the 'Avocet 2B x Avocet S' population segregated for a single dominant disease resistance gene that mapped to the Yr27 locus, (ii) all ten loss-of-function mutants had a non-synonymous polymorphism in the NLR gene, and (iii) the NLR gene was polymorphic between Avocet 2B and Avocet S, but identical between Avocet 2B and Kariega. In summary, we provide sufficient evidence that the cloned NLR corresponds to QYr.sgi-2B.1 from Kariega (=Yr27), which was the target of this gene cloning project.*

In Supplementary figure S3, the sample "Avocet 2B 24 hrs of inoculation has an incredible big error bar that makes a relative expression value of 4 not significantly different from the value of time zero (about 1). This experiment could be repeated.

*Our response: This experiment was repeated with five biological replicates and the supplementary figure (now Extended Data Fig. 4) replaced.*

Reviewer #2: Remarks to the Author:

A. A high quality genome assembly was created for the wheat cultivar Kariega, which has durable resistance to yellow rust. The durable resistance is the result of stacking of multiple resistance genes in the line. The QTL, which is responsible for some resistance, QYr.sgi-4A.1 was isolated in a recombinant population and found to provide resistance to one rust isolate (6E22A+), so the gene only accounts for one race in the durable resistance profile. The candidate gene was provided by homology of several genes to NBS type resistance genes. EMS mutagenesis of a resistant line (Avocet B) resulted in ten mutants at one of the NBS genes, which was labeled Yr27. The gene is expressed, and expression is elevated upon inoculation in seedlings. Silencing of the gene also resulted in susceptible plants. The authors add the added finding that the gene is closely related to other resistance genes and extrapolate that small changes in coding of Yr27 should provide resistance against other pathogens.

B. The findings are significant in the dissection of durable resistance and a high quality map of hexaploid wheat. The identification of an NBS gene as a resistance gene is not novel but the work was well-done and constitutes considerable effort in the identification of the gene. The gene was not transferred to a susceptible line by gene transfer. Nonetheless, a variety of techniques were used to corroborate the identify of Yr27.
*Our response: While we agree that a role of NLR genes in plant immunity is well documented in literature, we are not aware of any other example where two highly similar alleles of a NLR gene provide race-specific resistance to two different pathogens. This finding thus provides novelty in our view.*

C. The data and methodology was well presented.

D. The use of statistics was appropriate.

E. The Results were straightforward and robust.

F. The paper would be extraordinary, if manipulation of the gene led to new resistance specificity rather than just postulated.
*Our response: The manipulation of Yr27 and Lr13 and the possible generation of new resistance specificities is definitely a very interesting long-term project. Despite the close relatedness of Yr27 and Lr13 this will not be a straightforward endeavor and, in our view, is not within the scope of this manuscript. However, we have taken into account reviewer 4's suggestion to model the Yr27 protein. This allowed us to make predictions about amino acid residues that might be critical for the interaction with an effector or a guardee. In particular, the highly polymorphic amino acid stretch in the LRR domain might correspond to the inner surface of the LRR domain that has been shown to determine effector recognition specificity (see new Extended Data Fig. 5).*

Some minor comments:
1. "private to Yr27" is regional use of English (line 109)

*<u>Our response:</u> We replaced "private" with "only found in".*

2. Why are amino acid stretches approximate (eg line 104)
*<u>Our response:</u> This is a valid comment and we have replace the "approximate" with the exact values, starting with the first polymorphic residue and ending with the last polymorphic residue of each stretch.*
3. Avoid injecting assumptions, eg "surpisingly" (line 96)
*<u>Our response:</u> The word "surprisingly" was deleted.*

G. The references were adequate.

H. The paper was clear. However, the conclusion that the results "show that highly similar alleles of single-copy genes can confer resistance to different pathogen, providing a basis for engineering immune receptors with multiple recognition specificities in future" contains a number of assumptions not supported by research and is largely speculative. The thought could be stated prior to the research given the wide range of NBS and RLK homologs with different specificities.
*<u>Our response:</u> We agree that the thought / idea of changing recognition specificities of NLR and RLK genes is not novel and has been proposed multiple times. Despite some promising research, however, progress on engineering new recognition specificities has been rather limited so far. As stated above, our discovery that two highly similar alleles provide race-specific resistance to two different pathogens is novel and might provide a novel lead to manipulate recognition specificities of Yr27/Lr13 in particular and of NLR genes in general. We have slightly adapted our concluding sentence and made it more specific to our results. The sentence now reads as follows (lines 25-29): 'Our results thus demonstrate the feasibility of generating chromosome-scale wheat assemblies to clone genes and also exemplify that highly similar alleles of a single-copy gene can confer resistance to different pathogens, which might provide a basis for engineering Yr27 alleles with multiple recognition specificities in future.'*

Reviewer #3:

Remarks to the Author:

The authors took the advantage of recently fast developing field in sequencing and bioinformatic analysis and cloned a gene Yr27 conferring stripe rust resistance in wheat. Interestingly, they were able to show that Yr27 and recently cloned Lr13 were allelic, however, conferred resistance to different pathogens. The manuscript was very clearly and well written and I enjoyed reading it. Even though the gene structure is not something novel, the manuscript presented a breakthrough in combining different techniques to get a good genome assembly within very short period of time for wheat, which has a large genome size of about 16Gb. Their manuscript also provided useful information on the techniques so that the other researchers working on wheat and other plant species can all benefit from.

After identifying the gene candidate with mutants, VIGS was used to confirm the candidate. Although stable transformation would be even better, the evidence provided in the manuscript was solid enough to validate the candidate.

Suppl. Fig. 3: Top panel, 35 day-old plants, there is a hike at 24hpi for Avocet 2B, which is very different compared to the rest. Any explanation?
Our response: *This time point showed a very large standard error and we have repeated this experiment as suggested by reviewer 1.*

Is it correct that 35 day-old plants are already at flag-leaf stage?
Our response: *Yes, this is correct. We use the 'speed breeding' conditions with prolonged day length and the plants reach flag-leaf stage after 35 days.*

For 35 day-old plants, the rust response for Avocet S does not seem right and is too low, which should be highly susceptible. Any explanation?
Our response: *For consistency across this experiment, leaves of all samples were harvested 15 days after inoculation. Stripe rust development in Avocet S can sometimes be slightly delayed in 35-day-old plants compare to younger plants, which is why the 35-day-old plants looked slightly different from the younger plants.*

There are some addtional points for the authors to attend to:

L40: There are current 83 catalogued Yr genes, instead of 78, please revise;
Our response: *done*

The Yr genes cloned: Yr5 (Yr5a), YrSP (Yr5b), Yr7, Yr15, Yr36; Yr18 and Yr46 in the complex; and three temporary designated genes Yr10cg, YrAS2388R and YrU1.
Our response: *The reference cited at this position lists Yr5 and YrSP (Yr5b) as alleles of the same gene, which is why we report nine cloned stripe rust resistance genes.*

L115: For the ~11-fold coverage, the N50 is close to 5.0 Mb instead of 4.2 Mb. Please check Fig. 2, the N50 curve.
Our response: *This is correct. The N50 increases almost exponentially between a 7-fold and 15-fold coverage. We have thus indicated the exact values (taken from Supplementary table 2) in the main text.*

Fig. 1g: "N895D" was actually magenta, not pink. Also change in the figure caption, from "highlighted" in brown or magenta to in brown or magenta font colour.
Our response: *changed as suggested*

L197: Better write as "R:S = 254:91, $\chi 2$ = 0.348, …".
Our response: *done*

L328: Cat. No. M0880 is enough for EMS, package size is not necessary.
Our response: *changed accordingly*

L375: Change to "Si3 (198bp) in CDS" to make it clearer.
Our response: *done*

Please update the references because several papers have been formally published and have page number now.
*Our response:* *done*

Suppl. Fig. 1: The green squares are not very visible, either change the colour or make the border thicker.
*Our response:* *This was an issue with the resolution in the Supplementary PDF file. We now provide all supplementary figures as high-resolution 'Extended Data'.*

Suppl. Tables 4 and 5: In the title, "tissues" are more appropriate than "developmental stages".
*Our response: changed as suggested*

Reviewer #4:

Remarks to the Author:

The authors demonstrate efficient cloning of disease resistance gene from wheat that was enabled by rapid construction of chromosome level genome assembly (14 Gb, polyploid) from long reads and other data. The study builds on breakthroughs in genome assembly methods and new and better sequencing technologies, yet it is not an incremental advance – the assembly authors present is many folds better than previous efforts and they demonstrate the feasibility and democratization of their approach surpassing the need to have international consortia. present a subsampling experiment to demonstrate assembly quality at different sequencing depth. Technically, the study is outstanding, and I have no further comments or concerns. The paper is very clearly written.

My main suggestion for authors is to provide more details on the identified gene Yr27. It is an NLR and there is nothing novel about it, yet there is a lot of underexplored biology in NLRs that can recognize different pathogens. Specifically, I would recommend illustrating natural diversity and critical variants using structural information for NB-ARC and LRR domain and place these amino acid changes in the functional context of the protein. Do amino acid polymorphisms in NB-ARC overlap with functional motifs? Do they cluster on the side of the domain that is in contact with the LRR and therefore controls receptor? Similarly, do LRR amino acid changes cluster on the concave side of the protein where a ligand would bind or are they likely in contact with NB-ARC? Is Yr27 a highly variable NLR based on its entropy footprint? A simple modeling with Phyre using newest available NLR structures can help to answer these questions and further enrich presented analyses.
*Our response:* *Thank you very much for this excellent suggestion. A Shannon entropy plot with various Yr27/Lr13 variants revealed a higher variability in the LRR region compared to the CC and NB-ARC domains. The amino acid stretch in the LRR that showed a high variability between Lr13 and Yr27 overlaps with a region of the Arabidopsis RPP1 NLR protein that is involved in the interaction with the ATR1 effector. In particular, the unique residue N-895 would be part of the inner surface of the LRR and could be involved in polar interactions between Yr27 and an effector protein according to this model. Together, these results indicate that the variable amino acid stretch in the LRR might be involved in determining binding specificity, possibly by directly*

*interacting with a corresponding effector protein. We have summarized these results in the new Extended Data Fig. 5 and in the main text as follows (lines 109-123): 'Analysis of Shannon entropy, an unbiased measure to predict rapidly evolving residues, revealed a higher level of amino acid diversity in the LRR domain among Yr27/Lr13 protein variants present in various wheat cultivars compared to the CC and NB-ARC domains (Extended Data Fig. 5a, b). Beside leaf rust resistance, Lr13 also results in hybrid necrosis in certain genetic backgrounds. Two amino acids in the NB-ARC domain (I-346, K-562) of Lr13 are critical for leaf rust resistance specificity. At the equivalent positions, Yr27 had the same residues as the Lr13_hb haplotype, which lacked resistance to leaf rust but retained the hybrid necrosis phenotype. A comparison with publicly available wheat sequences revealed one amino acid (N-895) that was only found in Yr27 (Fig. 1g), which we turned into a Yr27-specific molecular marker for breeding (Extended Data Fig. 5c). Protein modelling predicted that three of the polymorphic residues in the highly variable 159 aa region of the Yr27 LRR domain (including the unique N-895) coincide with the inner surface of the LRR domain (Extended Data Fig. 5d). The corresponding residues of the Arabidopsis thaliana NLR immune receptor RPP1 contribute to the interaction of RPP1 with the ATR1 pathogen effector. Together, these data might indicate that Yr27 is a highly variable NLR and that the variable stretch in the LRR domain might be important to define recognition specificity.'.*

**Decision Letter, first revision:**

Our ref: NG-LE57826R1

18th Nov 2021

Dear Dr. Krattinger,

Thank you for submitting your revised manuscript "Long-read bread wheat genome enables disease resistance gene cloning" (NG-LE57826R1). It has now been seen by the original referees and their comments are below. The reviewers find that the paper has improved in revision, and therefore we'll be happy in principle to publish it in Nature Genetics, pending minor revisions to satisfy the referees' final requests and to comply with our editorial and formatting guidelines.

We are now performing detailed checks on your paper and will send you a checklist detailing our editorial and formatting requirements soon. Please do not upload the final materials and make any revisions until you receive this additional information from us.

Thank you again for your interest in Nature Genetics Please do not hesitate to contact me if you have any questions.

Sincerely,
Wei

Wei Li, PhD
Senior Editor
Nature Genetics
New York, NY 10004, USA
www.nature.com/ng

Reviewer #1 (Remarks to the Author):

The authors have revised the manuscript according to the suggestion of the referee(s) and I support the publication of the work.

Reviewer #2 (Remarks to the Author):

The authors have toned down some of the claims in regards to the previous manuscript. The application of long read technology is a technical advance and was put to good use in this work. The identification of the R gene is interesting for an understanding of xo-called durable resistant genes. The MS could be further strengthens by extending the discussion of the basis of claims for durability of individual genes, for example Yr18, and the notion that durability may, in many cases, may be unknown stacked R genes.

On a minor note, the paper is largely written in the first person (our results, we crossed, we used) and uses a number of adjectives for which we have not defined scale. For example, very high quality as opposed to high quality? Is this defined?

The title should read "facilitates" disease resistance gene cloning rather than "enables". R genes have been cloned prior to this work.

Reviewer #3 (Remarks to the Author):

The three key achievements/findings of the manuscript are: Demonstrated that chromosome-scale wheat assemblies could be generated from any wheat line so that gene of interest can be cloned. The main advantage of this gene cloning method is that it does not need time-consuming and laborious genome complexity reduction as required by other methods, such as MutRenSeq and MutChromSeq.

The second finding is that Yr27 and recently cloned Lr13 are allelic. This is the first report in wheat at least showing that two highly similar alleles provide race-specific resistance to different pathogen species. At the equivalent positions, Yr27 had the same residues as the Lr13_hb haplotype, which lacked resistance to leaf rust but retained the hybrid necrosis phenotype.

The third one is gene-specific marker was developed from Yr27 sequence so that it can be used in marker-assisted breeding/selection. Gene-marker for adult plant resistance gene is particularly useful because it will facilitate the selection and crossing without growing plants to the adult plant stage, and hence, can save a lot of time. Therefore, this study also has a practical implication in wheat breeding in combining Yr27 with other genes.

The authors have responded to my questions well, so as to the other reviewers', and the manuscript has been further improved taking up the suggestions by the reviewers, with the gene expression experiment redone (Extended Data Fig. 4), Yr27 haplotype analysis and homology modelling added (Extended Data Fig. 5), and other changes.

The authors updated the infected Avocet S leaves at flag leaf stage in Extended Data Fig. 4, which look good now (the way they should be, susceptible).

L213: Should be "$\chi 2$ = 0.348", not "X2".

Reviewer #4 (Remarks to the Author):

The authors addressed all of my suggestions.

My only remaining comment is that authors might want to explore de novo structure prediction with AlphaFold and compare to homology-based model given that RPP1 is so evolutionary distant. This might provide a higher quality model based on our experience. However, given that homology model was sufficient placement of variable amino acids on concave side of LRR, current analyses address my question.

**Author Rebuttal, first revision:**

**Point-by-point responses (email from 15 December 2021)**

Reviewer #2:
Remarks to the Author:
The authors have toned down some of the claims in regards to the previous manuscript. The application of long read technology is a technical advance and was put to good use in this work. The identification of the R gene is interesting for an understanding of xo-called durable resistant genes. The MS could be further strengthens by extending the discussion of the basis of claims for durability of individual genes, for example Yr18, and the notion that durability may, in many cases, may be unknown stacked R genes.

*Our response:* *To address this point we have included the following statement (lines 136-138): 'The longevity of the rust resistances in Kariega and Forno might be the result of optimal 'stacks' of partial broad-spectrum resistance genes, such as Lr34/Yr18, and race-specific R genes, indicating that the optimal deployment of race-specific resistance genes, in combination with partial, broad-spectrum resistance genes, can result in near-complete and long-lasting disease resistance.'*

On a minor note, the paper is largely written in the first person (our results, we crossed, we used) and uses a number of adjectives for which we have not defined scale. For example, very high quality as opposed to high quality? Is this defined?

*Our response:* *We have removed the 'very' in lines 66 and 124 because they are not strictly defined.*

The title should read "facilitates" disease resistance gene cloning rather than "enables". R genes have been cloned prior to this work.

*Our response:* *Done*

Reviewer #4:
Remarks to the Author:
The authors addressed all of my suggestions.

My only remaining comment is that authors might want to explore de novo structure prediction with AlphaFold and compare to homology-based model given that RPP1 is so evolutionary distant. This might provide a higher quality model based on our experience. However, given that homology model was sufficient placement of variable amino acids on concave side of LRR, current analyses address my question.

*<u>Our response:</u> We used AlphaFold and the results were largely consistent with the homology-based model. In particular, the location of the three polymorphic residues in the inner surface of the LRR domain was predicted both by homology-based modelling and AlphaFold. At this stage, we cannot predict if one of the two approaches is of 'higher quality' and we decided to only include the homology-based model. As the reviewer stated, the homology model was sufficient to identify and place potential critical amino acids.*

**Final Decision Letter:**

25th Jan 2022

Dear Dr. Krattinger,

I am delighted to say that your manuscript "Long-read genome sequencing of bread wheat facilitates disease resistance gene cloning" has been accepted for publication in an upcoming issue of Nature Genetics.

Over the next few weeks, your paper will be copyedited to ensure that it conforms to Nature Genetics style. Once your paper is typeset, you will receive an email with a link to choose the appropriate publishing options for your paper and our Author Services team will be in touch regarding any additional information that may be required.

After the grant of rights is completed, you will receive a link to your electronic proof via email with a request to make any corrections within 48 hours. If, when you receive your proof, you cannot meet this deadline, please inform us at rjsproduction@springernature.com immediately.

You will not receive your proofs until the publishing agreement has been received through our system.

Due to the importance of these deadlines, we ask that you please let us know now whether you will be difficult to contact over the next month. If this is the case, we ask you provide us with the contact information (email, phone and fax) of someone who will be able to check the proofs on your behalf, and who will be available to address any last-minute problems.

Your paper will be published online after we receive your corrections and will appear in print in the next available issue. You can find out your date of online publication by contacting the Nature Press Office (press@nature.com) after sending your e-proof corrections. Now is the time to inform your Public Relations or Press Office about your paper, as they might be interested in promoting its publication. This will allow them time to prepare an accurate and satisfactory press release. Include your manuscript tracking number (NG-LE57826R2) and the name of the journal, which they will need when they contact our Press Office.

Before your paper is published online, we shall be distributing a press release to news organizations worldwide, which may very well include details of your work. We are happy for your institution or funding agency to prepare its own press release, but it must mention the embargo date and Nature

Genetics. Our Press Office may contact you closer to the time of publication, but if you or your Press Office have any enquiries in the meantime, please contact press@nature.com.

Acceptance is conditional on the data in the manuscript not being published elsewhere, or announced in the print or electronic media, until the embargo/publication date. These restrictions are not intended to deter you from presenting your data at academic meetings and conferences, but any enquiries from the media about papers not yet scheduled for publication should be referred to us.

Please note that <i>Nature Genetics</i> is a Transformative Journal (TJ). Authors may publish their research with us through the traditional subscription access route or make their paper immediately open access through payment of an article-processing charge (APC). Authors will not be required to make a final decision about access to their article until it has been accepted. <a href="https://www.springernature.com/gp/open-research/transformative-journals"> Find out more about Transformative Journals</a>

<B>Authors may need to take specific actions to achieve <a href="https://www.springernature.com/gp/open-research/funding/policy-compliance-faqs"> compliance</a> with funder and institutional open access mandates. For submissions from January 2021, if your research is supported by a funder that requires immediate open access (e.g. according to <a href="https://www.springernature.com/gp/open-research/plan-s-compliance">Plan S principles</a>) then you should select the gold OA route, and we will direct you to the compliant route where possible. For authors selecting the subscription publication route our standard licensing terms will need to be accepted, including our <a href="https://www.springernature.com/gp/open-research/policies/journal-policies">self-archiving policies</a>. Those standard licensing terms will supersede any other terms that the author or any third party may assert apply to any version of the manuscript.

Please note that Nature Research offers an immediate open access option only for papers that were first submitted after 1 January, 2021.

If you have any questions about our publishing options, costs, Open Access requirements, or our legal forms, please contact ASJournals@springernature.com

If you have posted a preprint on any preprint server, please ensure that the preprint details are updated with a publication reference, including the DOI and a URL to the published version of the article on the journal website.

To assist our authors in disseminating their research to the broader community, our SharedIt initiative provides you with a unique shareable link that will allow anyone (with or without a subscription) to read the published article. Recipients of the link with a subscription will also be able to download and print the PDF.

As soon as your article is published, you will receive an automated email with your shareable link.

You can now use a single sign-on for all your accounts, view the status of all your manuscript submissions and reviews, access usage statistics for your published articles and download a record of your refereeing activity for the Nature journals.

An online order form for reprints of your paper is available at <a href="https://www.nature.com/reprints/author-reprints.html">https://www.nature.com/reprints/author-reprints.html</a>. Please let your coauthors and your institutions' public affairs office know that they are also welcome to order reprints by this method.

If you have not already done so, we invite you to upload the step-by-step protocols used in this manuscript to the Protocols Exchange, part of our on-line web resource, natureprotocols.com. If you complete the upload by the time you receive your manuscript proofs, we can insert links in your article that lead directly to the protocol details. Your protocol will be made freely available upon publication of your paper. By participating in natureprotocols.com, you are enabling researchers to more readily reproduce or adapt the methodology you use. Natureprotocols.com is fully searchable, providing your protocols and paper with increased utility and visibility. Please submit your protocol to https://protocolexchange.researchsquare.com/. After entering your nature.com username and password you will need to enter your manuscript number (NG-LE57826R2). Further information can be found at https://www.nature.com/nprot/.

Sincerely,

Wei Li, PhD
Senior Editor
Nature Genetics
New York, NY 10004, USA
www.nature.com/ng

Click here if you would like to recommend Nature Genetics to your librarian
http://www.nature.com/subscriptions/recommend.html#forms

** Visit the Springer Nature Editorial and Publishing website at <a href="http://editorial-jobs.springernature.com?utm_source=ejP_NGen_email&utm_medium=ejP_NGen_email&utm_campaign=ejp_NGen">www.springernature.com/editorial-and-publishing-jobs</a> for more information about our career opportunities. If you have any questions please click <a href="mailto:editorial.publishing.jobs@springernature.com">here</a>.**
